# RdDM*-*independent de novo and heterochromatin DNA methylation by plant CMT and DNMT3 orthologs

**DOI:** 10.1038/s41467-019-09496-0

**Published:** 2019-04-08

**Authors:** Rafael Yaari, Aviva Katz, Katherine Domb, Keith D. Harris, Assaf Zemach, Nir Ohad

**Affiliations:** 1 School of Plant Sciences and Food Security, Tel-Aviv University, 69978 Tel- Aviv, Israel; 2 The Manna Center Program for Food Safety and Security, Tel Aviv University, 69978 Tel-Aviv, Israel

**Keywords:** Enzyme mechanisms, DNA methylation, Epigenomics, Plant evolution

## Abstract

To properly regulate the genome, cytosine methylation is established by animal DNA methyltransferase 3 s (DNMT3s). While altered DNMT3 homologs, *Domains rearranged methyltransferases* (*DRMs*), have been shown to establish methylation via the RNA directed DNA methylation (RdDM) pathway, the role of true-plant DNMT3 orthologs remains elusive. Here, we profile de novo (RPS transgene) and genomic methylation in the basal plant, *Physcomitrella patens*, mutated in each of its *PpDNMTs*. We show that PpDNMT3b mediates CG and CHH de novo methylation, independently of PpDRMs. Complementary de novo CHG methylation is specifically mediated by the CHROMOMETHYLASE, PpCMT. Intragenomically, PpDNMT3b functions preferentially within heterochromatin and is affected by PpCMT. In comparison, PpDRMs target active-euchromatic transposons. Overall, our data resolve how DNA methylation in plants can be established in heterochromatin independently of RdDM; suggest that DRMs have emerged to target euchromatin; and link DNMT3 loss in angiosperms to the initiation of heterochromatic CHH methylation by CMT2.

## Abstract (web summary)

Whether plants have true DNMT3 orthologs and their role in establishing DNA methylation are still unclear. Here, the authors show that DNMT3s are persistent through plant evolution and mediates both de novo and heterochromatin DNA methylation in the early divergent land plant *Physcomitrella patens*.

## Introduction

DNA methylation, the addition of a methyl group to a cytosine base, is a prominent epigenetic modification in many eukaryotes^1–5^. It is catalyzed by distinct DNA methyltransferase (DNMT) families of proteins that share a conserved methyl-transferase domain (MTD)^1,4,6,7^. In plants, DNMTs evolved to methylate cytosines located in specific contexts (CG, CHG, and CHH; H=A, C, or T), distinct genetic elements (e.g., transposons and genes), various chromatin configurations (hetero-chromatin and eu-chromatin), as well as to establish methylation de novo at unmethylated sites or to maintain methylation upon DNA replication^4,6,8–13^. Plants encode four types of DNMTs: Methyltransferase 1 (*MET1*), DNA methyltransferase 3 (*DNMT3*), chromomethylase (*CMT*), and domain rearranged methyltransferase (*DRM*)^4,8,14^. MET1s are homologs of mammalian DNMT1 and maintain CG methylation^4^. CMTs are plant specific DNMTs first to appear in charophytes^15^. *Arabidopsis thaliana* (*Arabidopsis*) CMT2 and CMT3 orthologs utilize their chromodomain (CD) to bind to histone H3 lysine 9 dimethylation (H3K9me2) heterochromatin and to methylate CHH and CHG sites, respectively^16–18^. DNMT3s are ancient DNMTs that exist in animals, plants, and other eukaryotes^1,19^. Mammalian DNMT3s function primarily as de novo CG methylases and in specific tissues also at CH sites^1,6,20^. However, despite their significant role in mammals, non-animal DNMT3s have not been investigated thus far. DNMT3s were overlooked in plants probably due to their deficiency in angiosperms (flowering plants) and the discovery of their close homologs, DRMs, which function in de novo methylation. DRMs are plant specific DNMTs with a rearranged DNMT3-MTD^21^. Angiosperm DRMs are a part of the RNA directed DNA methylation (RdDM) pathway that utilizes small RNA to establish de novo methylation within euchromatic transposons, that is enriched with active histone marks such as H3K4me3 and depleted of repressive marks as H3K9me2^22–25^. So far, the function of plant DNMTs was comprehensively investigated in *Arabidopsis thaliana* and partially explored in a few additional angiosperms, all of which lack DNMT3 in their genomes^4,8,26–29^.

Here, we investigate both de novo (transgene) and maintenance (whole genome) DNA methylation activities in the early divergent land plant, *Physcomitrella patens (P. patens)*, which encodes all four types of plant DNMTs, including two DNMT3s. These experiments reveal unique biosynthetic methylation mechanisms of plant DNMTs, which postulate the selection forces leading to the appearance and disappearance of specific methylation pathways during plant evolution.

## Results

### Plant DNMT3s are evolutionary distinct from DRMs

**Fig. 1:**
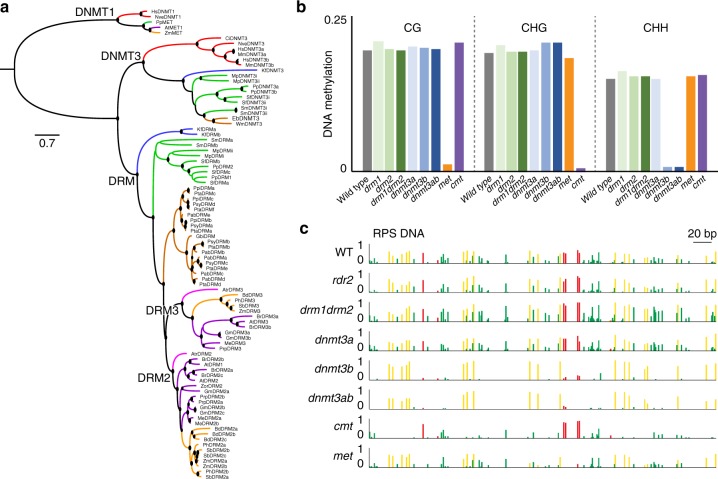
PpDNMT3b and PpCMT establish DNA methylation and maintain the entire non-CG methylome. **a** Sequences of DNMT3 and DRM MTD regions were aligned using MUSCLE^64^ (Supplementary Fig. 2). The phylogenetic tree was constructed by IQ-TREE^62,63^ and illustrated by FigTree. DRM MTDs were reorganized to fit the linear motif order as in canonical DNMTs. DNMT1 homologs were added as an outgroup. Clades having bootstrap value above 70% are marked with a circle. Protein accessions are listed in Supplementary Table 1. Colors depict taxonomic groups: red–animals; blue–charophytes; green–non-seed land plants; brown–gymnosperms; magenta–basal angiosperms; orange–monocots; purple–dicots. For alignment see Supplementary Fig. 1. **b** Averaged genomic cytosine methylation of WT and DNMT mutants in three sequence contexts, CG, CHG, and CHH. See Supplementary Table 2 for detailed information. **c** RPS methylation level (forward strand) in WT and indicated mutants. Red, yellow, and green represent CG, CHG, and CHH methylation, respectively. Source data of Fig. 1c is provided as a Source Data file

*P. patens* encodes two DNMT3s, designated here as PpDNMT3a and PpDNMT3b, which are composed of a DNMT3-type N-terminal MTD and a C-terminal domain of unknown function 3444 (DUF3444)^14^. Our genome and transcriptome searches revealed that this protein organization is conserved among non-flowering streptophytes DNMT3s (Supplementary Fig. 1). The existence of two full-length DNMT3 homologs (Supplementary Fig. 1) in two distantly-related gymnosperm subclasses that were separated around 300 million years ago implies upon the persistence of DNMT3 in gymnosperms. We did not detect DNMT3 in any available angiosperm genomes or transcriptomes, supporting the notion that DNMT3 completely disappeared from this plant lineage. Phylogenic analysis of the MTD showed that plant DNMT3 form a monophyletic clade together with animal DNMT3 which is separated from the DRM clade (Fig. 1a), suggesting the functional conservation of DNMT3s among plants and animals and/or functional speciation between plant DNMT3 and DRM proteins. Additionally, while DRM paralogs are common along plant evolution, they diverged into distinct orthologs only in seed plants, e.g., DRM2 and DRM3 in angiosperm (Fig. 1a), implying on further functional diversification of DRMs in this plant lineage. Paralogs of plant DNMT3s are also common, however based on our evolutionary analysis, these duplications did not evolve into conserved DNMT3 ortholog families across multiple species (Fig. 1a). Of note, PpDNMT3a and PpDNMT3b are not orthologs of mammalian DNMT3a and PpDNMT3b, respectively (Fig. 1a). Similarly, PpDRM1 and PpDRM2 are not orthologs of angiosperm DRM1 and DRM2, respectively (Fig. 1a). In summary, while DRMs are commonly considered as the plant homologs of eukaryotic DNMT3, here we show that DRMs are evolutionary distinct from DNMT3, and that true DNMT3 plant homologs exist throughout the plant kingdom, except in angiosperm.

### PpDNMT3b and PpCMT maintain the non-CG methylome

To determine the role of *P. patens* DNMTs in DNA methylation, we profiled the methylomes of *P. patens* DNMT deletion mutant plants, namely *met*, *cmt*, *dnmt3a*, *dnmt3b*, *drm*1, and *drm2* single deletion mutants, as well as in *drm1/drm2* (*drm1drm2*) and *dnmt3a/dnmt3b* (*dnmt3ab*) double deletion mutants^30,31^; (Supplementary Fig. 3). All single and double *DRM* and *DNMT3* mutants were viable and developed similarly to wild type (WT) (Supplementary Fig. 4). Genomic methylation averages clearly showed that CG, CHG, and CHH sites were nearly eliminated and specifically disrupted in *met*, *cmt*, and *dnmt3b* mutants, respectively (Fig. 1b). More precisely, *met* mutant lost 93% of CG methylation, *cmt* mutant lost 97% of CHG methylation, and *dnmtb* mutant lost 95% of CHH methylation (Fig. 1b and Supplementary Table 2). The *dnmt3ab* double mutant lost 95% of CHH methylation, which is comparable to the CHH loss in *dnmt3b* single mutant. Neither single (*dnmt3a*, *drm1*, or *drm2*) nor double mutant (*drm1drm2*) showed any significant global hypo-methylation in any of the sequence contexts (Fig. 1b). These complete and specific hypo-methylations in *P. patens* DNMT mutants led us to conclude that CG, CHG, and CHH contexts in *P. patens* are directly and primarily methylated by *PpMET*, *PpCMT*, and *PpDNMT3b*, respectively.

### De novo methylation is dependent on PpDNMT3b and PpCMT

Profiling genomic methylation in DNMT mutants refers mainly to DNA methylation maintenance activities. To evaluate the activity of *P. patens* DNMTs in de novo methylation, we introduced the repetitive DNA sequence (RPS) from *Petunia hybrida*^32–34^, uncommon to moss, into *P. patens*. DNA methylation analysis of RPS was conducted in the first transgenic generation (T1) and within the same transformed plant tissue. Using bisulfite sequencing, we found that RPS is methylated in WT cells in all three methylation contexts, CG, CHG, and CHH (Fig. 1c), implying on its ability to be de novo methylated in *P. patens*. By introducing and examining RPS methylation in the various DNMT mutants, we found that CG methylation is significantly reduced in *met*, *dnmt3b* and *dnmt3ab* (paired t-test p-value <0.0016, 0.0023, 0.0022, respectively), CHG methylation is specifically and significantly reduced in *cmt* (paired t-test p-value <10^−5^), and CHH methylation is eliminated in *dnmt3b* and *dnmt3ab* mutants (paired t-test p-value <10^−5^ for both) while unchanged in *dnmt3a*. In *drm1drm2* mutant, RPS was methylated same as in WT. In angiosperms, DRMs are directed to the DNA by 24nt small interfering RNA (siRNA)^23^. Accordingly, we also tested the ability of RPS to be de novo methylated in a *P. patens* plants mutated in the *RNA Directed RNA polymerase 2 (PpRDR2)* and subsequently depleted of siRNA^35^. Similarly to *drm1drm2*, we found that RPS is regularly methylated in *P. patens rdr2* mutant plants. Altogether, these context-specific RPS methylation phenotypes in each of the mutants suggest that de novo methylation in *P. patens* can be mediated by *DNMT3b* at CG and CHH sites and by *CMT* at CHG sites without the involvement of DRMs or the canonical RdDM pathway. The reduction of CG methylation in RPS DNA in *met* T1 plants suggests that de novo CG methylation of RPS is relied also on PpMET. This assumption is in confirmation with published de novo methylation activity of mammalian *DNMT1* and *Arabidopsis* MET1 either by themselves or in cooperation with de novo methylases^36–38^. Alternatively, CG hypomethylation in *met* mutant could suggest that CG methylation in RPS is dependent on PpMET maintenance activity within just a few rounds of somatic cell generations.

### PpDNMT3b and PpCMT affect genomic CG methylation

**Fig. 2:**
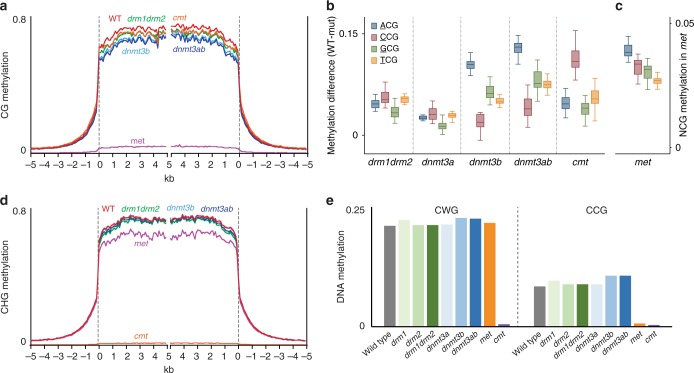
Genomic CG methylation regulation by PpCMT and PpDNMT3b. **a** Patterns of TE CG methylation in WT and indicated mutants. *P. patens* TEs were aligned at the 5′ end and average methylation for all cytosines within each 100 bp interval is plotted. The dashed lines represent the points of alignment. **b** Box plot of NCG methylation difference in TEs between WT and indicated mutants (N = any nucleotide). **c** Box plot of the residual NCG methylation in TEs in *met* mutant. Boxplots throughout the manuscript show the interquartile range (IQR) (box), center lines represent the median, and the whiskers corresponding to 1.5 times the IQR. **d** Patterns of TE CHG methylation in WT and indicated mutants (similar to B). **e** Averaged genomic CHG methylation level in WT and DNMT mutants separated to CWG (i.e., CAG or CTG) and CCG

The near-complete elimination of CG methylation in the *met* genome (Fig. 1b) suggests that unlike animal DNMT3, PpDNMT3s do not have a role in maintaining genomic CG methylation. However, by focusing on transposable elements (TEs), we found a consistent decrease of 13% in CG methylation in both single *dnmt3b* and double *dnmt3ab* mutants (Fig. 2a), suggesting that DNMT3b is partially involved in maintaining the CG methylome. Further dissection of CG methylation based on their neighboring 5′ nucleotides, i.e., NCG sites (N = any nucleotide), revealed that ACG sites are preferentially hypomethylated in *dnmt3b* and *dnmt3ab* (Fig. 2b). In association with the particular ACG hypo-methylation in *dnmt3b* plants, we found that in *met* mutant ACG sites exhibit the highest residual CG methylation levels (Fig. 2c).

Among the four NCG sites, CCGs had the lowest CG-hypomethylated effect in *dnmt3b* mutant (Fig. 2b). CCG is one form of CHG for which we have previously showed that its methylation (mCCG) in the entire *Arabidopsis* genome and a couple of examined sequences in *P. patens*, is dependent on the methylation of the internal CG site (CmCG) maintained by MET1 genes^31^. Here, we extended this observation to the entire *P. patens* genome by showing that CHG methylation, specifically at CCG sites, was diminished in the *met* mutant (Fig. 2e). This contributed to a 13% reduction in CHG methylation at TE sequences (Fig. 2d). Interestingly, we found that the reciprocate effect also exists, i.e., CmCG dependency on mCCG. Out of the four NmCG methylation contexts, CmCG was particularly reduced in the *cmt* mutant (Fig. 2b), while in *met* mutant CmCG residual level was second to ACG (Fig. 2c). Accordingly, along with their de novo methylation activities these results demonstrate the ability of PpCMT and PpDNMT3b in establishing CG methylation at genomic CCG and DCGs (D = A, G, or T) sites, respectively.

Non-CG methylation by mammalian DNMT3 is targeted preferentially to CW sites (W = A or T), such as CAC and CAG^20^. Herein we found CHH methylation (mediated by PpDNMT3b) to be preferentially targeted to CWH sites (Supplementary Fig. 5), suggesting for functional conservation of CW methylation between mammalian and moss DNMT3s. However, the particular regulation of CHG methylation (including of CWG) by PpCMT (Fig. 2e), infer on diversification of PpDNMT3b by avoiding methylating CWG sites that are controlled solely by PpCMT.

### PpDNMT3b mediates heterochromatic-mCHH and affected by PpCMT

**Fig. 3:**
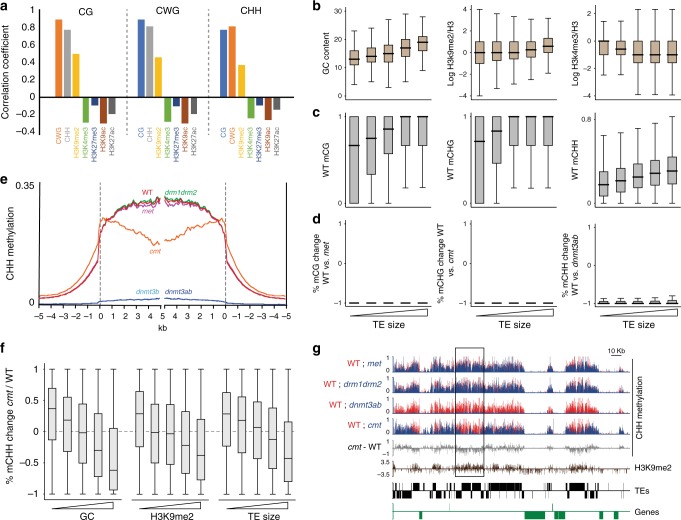
PpCMT and PpDNMT3 methylate heterochromatin. **a** Pearson correlation coefficients between CG/CHG/CHH methylation, GC content, and indicated histone modifications of TEs in 50 bp windows. **b** Box plots showing GC content, H3K9me2, and H3K4me3 levels in 50 bp windows within five quantile TE sizes. **c** Box plots of averaged DNA methylation in 50 bp windows of WT protonoma over five quantiles of TE sizes. **d** Box plots of percent-methylation-change between WT and indicated mutants 50 bp windows with a minimum 10% methylation in either of the samples, over TE size. **e** Patterns of TE CHH methylation in WT and indicated mutants as described in Fig. 2a. **f** Box plots showing the distribution of percent-methylation-change per 50 bp windows between WT and cmt mutant over H3K9me2, GC content, and TE size quantiles. **g** CHH methylation level (red WT, blue mutant), CHH methylation difference (cmt minus WT), H3K9me2, and gene/TE annotations of a representative region from Chromosome 1:459,000–702,000. Genes and TEs oriented 5′ to 3′ and 3′ to 5′ are shown above and below the line, respectively. Open black box marks a cmt hypo-methylated region enriched for H3K9me2

DNA methylation in *P. patens* is specifically targeted to TEs (Supplementary Fig. 6) and segregated away from genes^5^. Only about 0.5% of the methylated cytosines reside within genic sequences, which are mostly transcriptionally silenced^39^ and are controlled by PpDNMTs similarly to the way TE methylation is regulated by PpDNMTs (Supplementary Fig. 6). In agreement, DNA methylation in *P. patens* is positively associated with heterochromatic (i.e., H3K9me2) and negatively associated with euchromatic (e.g., H3K4me3) marks (Fig. 3a)^39,40^. We further showed that similarly to *Arabidopsis*, long TEs in *P. patens* tend to be more heterochromatic, whereas short TEs are more euchromatic (Fig. 3b)^18^. Consistent with the relationship with heterochromatin, we found DNA methylation level to associate with TE size, i.e., to accumulate at relatively longer TEs (Fig. 3c). These correlations of DNA methylation with heterochromatin, together with the complete or near complete elimination of CG, CHG, and CHH methylation in *met*, *cmt*, and *dnmt3b* mutants (Figs. 1b, 3d), respectively, suggest that PpMET, PpCMT and PpDNMT3b function preferentially within heterochromatic TE sequences.

Interestingly, while genome wide CHH methylation in *P. patens cmt* mutant was similar to levels in WT (Fig. 1b), when profiling methylation along TEs, we found that CHH methylation in *cmt* was substantially altered, i.e., increased closer to TE-edges and gradually decreased inward into the elements (Fig. 3e). In the TE meta-analysis short and long TEs were relatively enriched and depleted closer and away to the points of TE-alignment, respectively (Supplementary Fig. 7a). Consequently, we found that CHH methylation in *cmt* is preferentially decreased in long TEs and hyper-methylated in short ones (Fig. 3f). In accordance with the association of TE size with chromatin configuration (Fig. 3b), we found that CHH methylation in *cmt* mutant was preferentially depleted at genomic regions enriched for GC nucleotides and H3K9me2, and particularly increased within low GC and H3K9me2 TE regions (Fig. 3f, g). When focusing on short TEs (<500 bps), we found that hyper-methylation and hypo-methylation in *cmt* background continued to associate with eu-chromatic and hetero-chromatic regions, respectively (Supplementary Fig. 7b), suggesting that the chromatin structure, rather than TE size, determines the CHH methylation effect in *cmt* mutant. Overall, our results suggest that PpMET, PpCMT and PpDNMT3b function preferentially at H3K9me2-heterochromatic regions, and that PpDNMT3b CHH methylation activity is affected by PpCMT.

### PpDRMs target transcribed-euchromatic TEs

Neither single (*drm1* or *drm2*) nor double (*drm1drm2)* mutant showed reduction of global genomic methylation (Fig. 1b and Supplementary Fig. 8a). Similar to *drm* mutants, no effect on methylation was recently reported for *P. patens rdr2* mutant^35^, which we validated here while substantially expanding our analysis to a larger genomic portion (80 vs. 20%; Supplementary Fig. 8a and Supplementary Table 2). These results, together with the complete CG, CHG, and CHH hypo-methylation in *met*, *cmt*, and *dnmt3b* mutants, respectively (Fig. 1b), imply a trivial methylation activity of DRMs and RDR2 in *P. patens*.

**Fig. 4:**
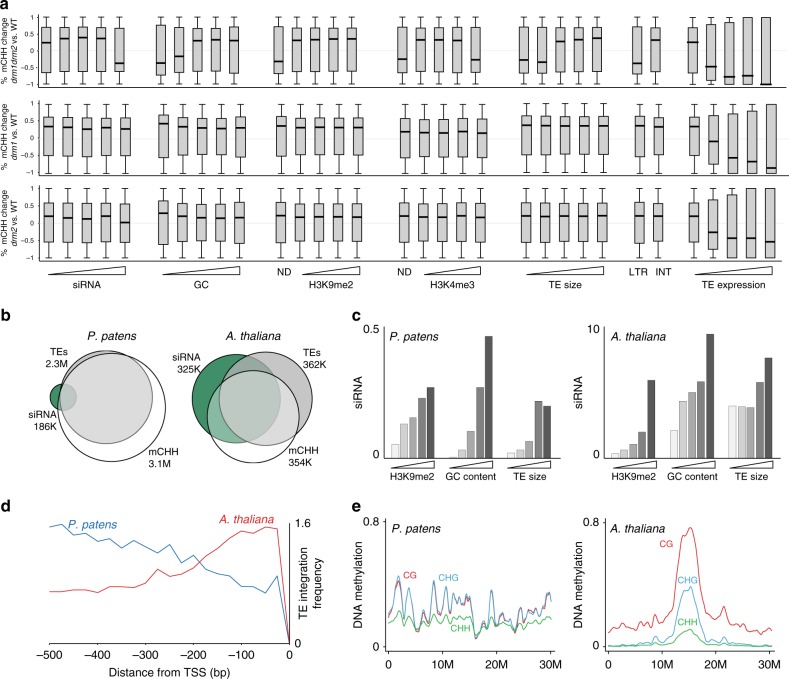
PpDRMs methylate active-euchromatic TEs. **a** Box plots of percent-methylation-change between indicated samples within differentially CHH methylated 50 bp windows, separated based on the level of various genomic/chromatin attributes. Note the hypo-methylation trend in protonema *drm1drm2* sample (top track) in genomic regions with high siRNA counts, low GC content, absent H3K9me2 signal, high H3K4me3 signal, short TEs, LTR annotations, and TE expression. **b** Venn diagram showing abundance and overlap between siRNA, CHH methylation, and TE annotation, in *Arabidopsis* and *P. patens*. **c** siRNA abundance over increased quantiles of indicated chromatin features in *A. thaliana* (**c**) and P. patens (**c**). **d** Patterns of TE integration in *Arabidopsis* and *P. patens* upstream to gene TSS. *Arabidopsis* or *P. patens* genes were aligned at the 5′ end (0 at *x* axis) and percentage of the number of TEs (first and closest nucleotide of TE to TSS) within each 25 bp is plotted. **e** LOWESS fit of DNA methylation distribution averaged in 100 kb bins across chromosome 1 in *Arabidopsis* and *P. patens*

As opposed to a global methylation phenotype, we next checked for a localized methylation effect in *drm* mutants within statistically supported differentially methylated regions (DMRs) separated into distinct chromatin configurations. While hypo-methylated DMRs were not significantly enriched over hyper-methylated DMRs in neither *drm* nor *rdr2* mutant (Supplementary Fig. 8b), we found that CHH-DMRs of *drm1drm2* double mutant were particularly hypo-methylated within genomic regions enriched for siRNA, low GC content, low histone H3 abundancy, high H3K4me3, short TEs, and long-terminal-repeat (LTR) regions of retrotransposons (Fig. 4a – top panel). Single mutants *drm1* and *drm2* CHH-DMRs were mostly hyper-methylated and did not associate with any chromatin or DNA features (Fig. 4a lower panels), thus implying for an unrelated noise, which is a common feature of asymmetric methylation. Under this assumption, the particular hypomethylation effect in *drm1drm2* (Fig. 4a top panel) suggests for some functional redundancy between DRM1 and DRM2. Intriguingly, we found CHH-DMRs of single and double *drm* mutants, as well as of *rdr2* to be gradually hypo-methylated within a small number of windows ( < = 5610) of expressed TEs (Fig. 4a right panels and Supplementary Fig. 8c, d), which were also abundant in H3K4me3 and depleted of H3K9me2 (Supplementary Fig. 8e). Overall, these results associate PpDRMs methylation activity with RDR2 generated siRNA, as well as with actively-transcribed euchromatic TE sequences, both of which are signatures of RdDM activity in angiosperm.

The weak genomic methylation activity of DRMs in *P. patens* could be explained by the exceptionally high efficiency of PpCMT and PpDNMT3b. PpCMT targets CHG methylation as strongly as PpMET targets CG methylation (Figs. 2a, d and 4e), and PpDNMT3b targets CHH methylation with more than twice the level of CHH methylation in *Arabidopsis* (Fig. 4e)^41^. Consequently, together with their ability to de novo methylate DNA, it is possible that PpCMT and PpDNMT3b target and maintain non-CG methylation even within euchromatic regions that have a weak heterochromatic signal.

In support of a trivial role for RdDM in *P. patens*, we found siRNA in *P. patens* to overlap with only 5% of methylated TEs, in comparison to 65% in *Arabidopsis* (Fig. 4b). Moreover, similarly to *Arabidopsis*, we found siRNA in *P. patens* to be enriched within long-heterochromatic TEs (Fig. 4c). In *Arabidopsis*, RdDM functions mostly in euchromatic TEs, while heterochromatic siRNAs are hardly involved in maintaining DNA methylation^18,42,43^. If the same is true in *P. patens*, then the exceptionally low abundance of siRNA in euchromatic TEs (0.9%) could further explain the minor role of PpDRMs in genomic methylation.

In addition to actively transcribed TEs, another source for euchromatic TEs could be those located in gene promoters^18,44^. Notably, we found that the frequency of TE integration within the first 200 bp upstream to transcription start site (TSS) of genes, was lower by up to 2.3 times in *P. patens* than in *Arabidopsis* (Fig. 4d). This result is counterintuitive, considering that the *P. patens* genome contains eight times more TEs than that of *Arabidopsis*, which are also spread more evenly along the chromosomes in comparison to the centric concentration of TEs in *Arabidopsis*^39^. Hence, the particular depletion of TEs in *P. patens* from gene promoters, which are known to be the main target of DRMs and RdDM in angiosperms^18,44–46^, could contribute for the weak genomic methylation effect of PpDRMs and RDR2 in *P.patens*.

## Discussion

**Fig. 5:**
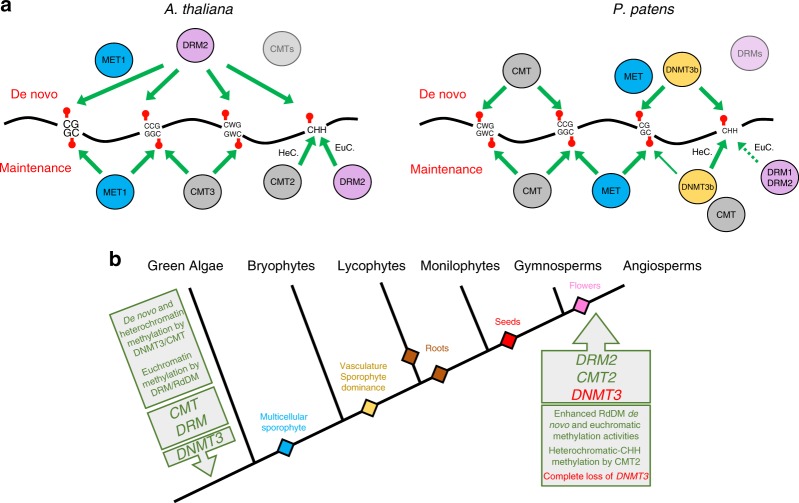
Mechanisms and evolution of plant DNMTs. **a** DNMT methylation mechanisms are illustrated based on current knowledge. Black line represents the DNA with different cytosine subcontexts embedded in it. Lollipops represent methylation. Arrows width is corresponding qualitatively to the relative level of methylation mediated by indicated DNMTs. HeC. = heterochromatin, EuC. = euchromatin. De novo and maintenance methylation activities are shown above and below the DNA, respectively. De novo methylation in *P. patens* is based on our RPS transgene results. Future studies would need to check the de novo methylation activity of CMTs and DRMs (masked ovals) in *Arabidopsis* (angiosperms) and *P. patens* (basal/DNMT3-encoding plants), respectively. **b** Schematic illustration of the evolution of plant DNMTs and their function based on previous and our studies. Backbone of phylogenetic tree is inspired by https://langdalelab.com/

To date, functional analyses of plant DNMTs were focused primarily on *Arabidopsis* and a few additional angiosperms. *P. patens* is a basal land plant that diverged from angiosperms about 400 million years ago^47^ and encodes homologs of all four plant DNMT protein families^14^, including DNMT3 which has been lost during angiosperms evolution. Thus, our comprehensive analysis of the entire PpDNMT proteins under de novo and homeostasis methylation conditions allowed us to reveal their function, as well as to infer on the evolutionary mechanisms of DNA methylation in plants (Fig. 5).

Mammalian DNMT3s function primarily as de novo methylases of CG sites and in some tissues also of CH sites^20^. We show here that PpDNMT3s are required for de novo methylation of CG and CHH sites (Fig. 1b). As PpDNMT3b is the first non-animal DNMT3 to be functionally characterized, our results imply that de novo methylation of CG and non-CG sites is an ancient feature of eukaryotic DNMT3 that predates the divergence of plant and animal DNMT3s. Additionally, our data demonstrate the ability of DNMT3 to specialized in their hosts, such as the preference of mammalian DNMT3 towards CG sites and that of moss DNMT3 towards CHH sites. Conservation and diversification between mammalian and moss DNMT3s would provide the basis for further structure-function interactions of eukaryotic DNMT3. The narrow overlap of siRNA with DNA methylation (Fig. 4b) and the trivial methylation effect in *Pprdr2* (Fig. 1c and Supplementary Fig. 8a), suggest that the robust genomic methylation of PpDNMT3 does not involve the RdDM pathway. In comparison, the association between PpDRMs methylation effect, siRNA signal (Fig. 4a), and PpRDR2 methylation profile (Supplementary Fig. 8d) link basal DRMs with RdDM. Consequently, these results suggest that since its emergence RdDM included DRMs rather than DNMT3s as its methylase component (Fig. 5b).

RPS methylation by PpCMT (Fig. 1c) is an in vivo evidence for de novo methylation by a CMT protein. In vitro studies have shown that *Arabidopsis* CMT2 and CMT3 can methylate unmethylated-DNA templates^16,17^. Thus, it is possible that CMTs in *Arabidopsis* and other angiosperms are capable of mediating de novo methylation, as well (Fig. 5a). CMT de novo methylation activity would help in resolving how DNA methylation is targeted to regions that are normally not regulated by RdDM, such as heterochromatic TEs and intra-genic sequences (gene bodies), methylation of the latter was recently genetically linked to CMT3^48^. Our de novo methylation data also support previous findings showing the ability of plant MET1s to methylate unmethylated CGs de novo or to reinforce it with other de novo methylases^37,38^ (Figs. 1c and 5a).

The antagonistic CHH methylation changes in *Ppcmt*, from hypomethylation in heterochromatin to hypermethylation in euchromatin (Fig. 3e–g), resembles the methylation phenotype of *Arabidopsis* histone *h1* mutation^18^. Similar to *Arabidopsis h1*, the elimination of CHG methylation in *Ppcmt* could disturb the chromatin in a way that affected regular CHH methylation activities, as well as demethylation ones^18,49^. Our data suggest that the role of H3K9me2 in targeting non-CG methylation in angiosperms^4,8^ has already been established in basal plants. However, unlike many angiosperms that utilize two CMT orthologues to methylate distinct non-CG contexts, i.e., CMT2 for CHH and CMT3 for CHG, basal plants use CMT for CHG and DNMT3 for CHH sites. Similar to angiosperm-CMTs, early diverged CMTs, such as PpCMT, probably also utilize their chromodomain to be targeted to H3K9me2-chromatin. Plant DNMT3s are missing a chromodomain, thus it is likely that the association of their CHH methylation with H3K9me2 is indirect. Mammalian DNMT3s were found to bind H3K9-methylated chromatin via attachment to chromodomain proteins or via unmethylated-H3K4 residues (H3K4me0), a histone mark associated with H3K9me2^7^. The partial dependency of CHH methylation on PpCMT/CHG methylation (Fig. 3f) and the absence of the reverse effect, i.e., control of CHG methylation by DNMT3/CHH methylation (Fig. 1b), suggest a hierarchy between CHG and CHH methylation. In this hierarchy, PpCMT is positioned on the higher level, possibly by recruiting the DNMT3 protein itself or by regulating the level of DNMT3 substrates, e.g., H3K9me2 and/or H3K4me0. An alternative explanation for the CHH hypomethylation in *cmt* (Fig. 3e), could be that PpCMT is involved in establishing CHH methylation that is subsequently maintained by PpDNMT3b. This hypothesis is supported by the ability of *Arabidopsis* CMTs to establish CHH methylation *in vitro*^16,17^, and by the residual of CWA methylation in *Ppdnmt3b* mutants (Supplementary Fig. 5) that resembles the preference of some angiosperm CMTs toward such CHH subcontext^50^.

*DRMs* most likely evolved from plant-*DNMT3s* (Fig. 1a). Additionally, thus far there is not a single plant species (including early-diverged ones) that encode a *DRM* as its only DNMT besides MET1. Therefore, our findings of de novo methylation by PpCMT and PpDNMT3b, suggest that de novo methylation in early diverged plants was dependent on DNMT3 and/or CMTs (Fig. 5b). Assuming that basal-DRMs inherited their ability for de novo methylation from their ancestral plant-DNMT3s, one could ask what the selective force for the appearance of DRMs was, considering that plants already had the machineries for de novo methylation of CG and non-CG sites (Fig. 5). We propose that DRM’s ability to mediate methylation to a particular chromatin region played an important role in their evolution. CMT and DNMT3 evolved to target H3K9me2-heterochromatin, which consequently induced the emergence of DRMs to function in euchromatin, which is depleted of H3K9me2 and enriched for H3K4me3. H3K4 methylation was shown to inhibit mammalian DNMT3^4^, and PpDNMT3′s CHH methylation activity is negatively associated with H3K4 methylation (Fig. 3a–d). Accordingly, the association of PpDRMs CHH methylation at H3K4me3-euchromatic sites (Fig. 4a and Supplementary Fig. 8e), suggests that DRMs evolved to target a specific type of nucleosomal DNA (i.e., containing H3K4me) that cannot be targeted directly by DNMT3. H3K4me3-associated euchromatic TEs include actively-transcribed TEs, as well as TEs that are located near genes^17,51,52^. Such genetic elements are required to be silenced for the benefit of the host. For that reason, DRMs and the RdDM pathway probably persisted along plant evolution, even in species such as *P. patens* with highly efficient DNMTs that could mediate methylation even to sequences with low heterochromatic signal (Fig. 3b–d). In seed plants, de novo and maintenance of genomic methylation by RdDM is evidently enhanced^53,54^, possibly in response to bursts of particular TE families that escaped silencing or integrated next to genes (Fig. 4d). The profound role of RdDM in angiosperms is associated with gaining additional genetic components that could have enhanced or expanded various steps in the pathway, such as siRNA biogenesis and methylation activity^53,54^. For example, the appearance of SHH1 in angiosperms, which binds H3K9me2 and required for siRNA biogenesis, may have expanded the activity of RdDM in these species towards more heterochromatic elements^55^. In addition to the developments in RdDM pathway, flowering plants also evolved a new CMT protein clade, that is CMT2, which targets heterochromatic CHH methylation via direct binding to H3K9me2^17,18^. Consequently, the enhancement of de novo and genomic methylation activity by DRMs and heterochromatic CHH methylation by CMT2 could have compensated for the primary activities of DNMT3s, which allow their complete extinction in angiosperm (Fig. 5b).

## Methods

### Biological materials

All mutant plants were generated in the background of ‘Gransden 2004’ strain of *P. patens*^47,56^ and were propagated on BCD or BCDAT media^57^ at 25 °C under a 16 h light and 8 h dark regime^58^.

### Generation of transgenic mutant lines

*P. patens* single deletion mutant lines for the following genes: *PpDRM1* (Pp3c15_14360V1.1), *PpDRM2* (Pp3c15_21430V1.1), *PpDNMT3a* (Pp3c3_3540V1.1) and *PpDNMT3b* (Pp3c13_8320V1.1) were generated by replacing the genomic region coding for the methyltransferase domain with either the hygromycin resistance cassette (*hptII*) or the G418 resistance cassette (*nptII*) via homologous recombination (illustrated in Supplementary Fig. 3). Genomic fragments corresponding to the 5′ and 3′ flanking regions of the deleted sequence were amplified using KOD hot start DNA polymerase (Novagen), cloned into the pTZ57 vector (Fermentas) and sequenced to validate their integrity. Next, the 5ʼ and 3' fragments were subcloned into either the pMBL5 vector (GenBank: DQ228130.1) or the pMBL5 Nos Hyg vector^31^. Constructs were introduced into protoplasts via PEG-mediated transformation as described^57^ using 15 µg of plasmid restricted to linearize the construct. Six days after regeneration, transformants were selected on BCDAT medium containing 25 µg/ml hygromycin (Duchefa) or 25 µg/ml G418 (calbiochem). Resistant plants were further tested by tissue PCR^31^ to verify correct integration of the construct into the genome, by amplifying the junction regions between the insert and the sequence flanking the deleted fragment at both the 5′ and 3′ ends (primers listed in Supplementary Table 2). In addition, loss of the endogenous targeted loci was correlated with lack of amplification of the targeted sequence as compared to a positive control*. ΔPpdrm2* and *ΔPpdnmt3a* single deletion mutant protoplasts were used to generate *ΔPpdrm1ΔPpdrm2* and *ΔPpdnmt3aΔPpdnmt3b* double deletion mutant lines, respectively, as described above.

### Generation of RPS transgenic lines

The RPS transgene was introduced into the genome of WT and mutant plants via non-homologous recombination. To this end, a pMBL5 + Zeo vector was constructed by subcloning the Zeocin resistance cassette (*Sh ble* gene) from pRT101-Zeo^33^ replacing the G418 resistance cassette (*nptII* gene) of the pMBL5 vector (GenBank: DQ228130.1). The RPS fragment was subcloned from the p35 GUS/RPS vector^33^ into pMBL5 + Zeo vector. Both the RPS and Zeocin resistance cassettes were sequenced in the final pMBL5 + Zeo + RPS construct to ensure integrity. Following transformation (as described above) and selection on BCDAT medium containing 50 µg/ml Zeocin (Invivogen), resistant plants were tested to verify insertion of the construct into the genome by tissue PCR^31^ amplifying an internal transgene sequence spanning both the RPS sequence and the selection cassette (primers listed in Supplementary Table 3).

### Validation of RPS absence in *P. patens* genome and sRNAome

The RPS sequence (GenBank: X92381.1) was used for homology search (blastn) in the *P. patens* V3.0 genome^59^. Additionally, it was used to search for corresponding small RNAs by NCBI SRA-Blast^60^ using small RNA-seq data of *P. patens* protonema^35^ (SRX247005-SRX247008 and SRX327325-SRX327330).

### Published genomic data

Data for sRNA were derived from^35^, for mRNA from^5^, and for histone modifications from^40^.

### Bisulfite sequencing of the RPS transgene

A fragment of RPS was PCR amplified from bisulfite treated genomic DNA, extracted from protonema tissue, using primers RPS-top-R-new and RPS-top-F (primers listed in Supplementary Table 3) and KAPA HiFi Uracil + polymerase (kappa biosystems), then cloned into pJET1.2 (Thermo Fisher Scientific). The methylation status of RPS forward strand of individual clones was determined by Sanger sequencing.

### Phylogenetic analysis

PpDNMT3b and PpDRM2 protein sequences were used to search for homologs by blastp vs. NCBI Non-redundant protein database^61^ and by tblastn vs. the 1000 plants (1kp) transcriptome database^62,63^. Alignment of selected DNMT3, DRM and DNMT1 MTD protein sequences was performed using MUSCLE v3.8.31^64^. The motif order was rearranged in DRM sequences to match the linear organization of canonical DNMTs. Protein accessions are listed in Supplementary Table 2. MTDs of animal and plant DNMT1/MET1 homologs were added as outgroup. The phylogenetic tree was constructed by IQ-TREE v1.6.4^65–67^ using default parameters and illustrated by FigTree v1.4.3 (http://tree.bio.ed.ac.uk/software/figtree/).

### BS-seq library preparation

Around 0.5 μg of genomic DNA from protonema tissue was extracted,sheared (by sonication), end repaired (10 µl T4 DNA ligase buffer (NEB B0202S), 4 µl 10 mM dNTP mix, 1 µl T4 DNA polymerase (NEB M0203S), 1 µl Klenow DNA polymerase (NEB M0210S), 1 µl T4 PNK (NEB M0201S), water to 100 µl), A-tailed (5 µl Klenow buffer (NEB2), 10 µl 1 mM dATP, 1 µl Klenow exo minus (NEB M0212S), water to 50 µl), and ligated to methylated-adapters (25 µl quick ligase buffer (NEB), 1 µl 10 mM preannealed bs-seq-adapters (Supplementary Table 2), 1 µl DNA quick ligase (NEB M2200S), water to 50 µl). Adaptor-ligated libraries were subjected to two sequential treatments of bisulfite conversion using the EpiTect Bisulfite kit (Qiagen). Bisulfite-converted libraries were amplified by PCR (2.5 U of ExTaq DNA polymerase (Takara Bio), 5 µl of 10x Extaq reaction buffer, 25 mM dNTPs, 1 µl bs-seq-primers (Supplementary Table 2), and adding water to 50 µl). PCR program: 95 °C for 3 min, then 12–14 cycles of 95 °C for 30 s, 65 °C for 30 s, and 72 °C for 60 s. Between library preparation steps and following PCR, DNA was purified with the solid-phase reversible immobilization method using AM-Pure beads (Beckman Coulter) and quantified with Bioanalyzer (Agilent). Deep sequencing was performed on Illumina Hi-Seq 2000.

### BS-seq data analysis

BS-seq reads were processed by converting all the Cs in the ‘forward’ reads to Ts, and all the Gs in the ‘reverse’ reads to As. Converted reads were aligned to the converted reference scaffold using Bowtie1^68^. Methylation level for individual cytosines along the chromosomes was calculated by counting the number Cs divided by the number of (C + T) (‘single-c’ files on GSE118153). Genomic methylation averages (Fig. 1b, Fig. 2e, Supplementary Fig. 4, and Supplementary Table 3) were calculated by averaging methylation of the entire single-c files separated to distinct sequence contexts (e.g., CG, CHG, or CHH) and nuclear vs. organelle chromosomes. Single-c files were further used to plot methylation patterns in TEs (Fig. 2a, Fig. 2d, and Fig. 3e), as well as fractional methylation within a 50 bp sliding window (‘w50’ files on GSE118153) that were used in downstream analyses (e.g., Fig. 2b, c, Fig. 3a, c, d, and f, and Fig. 4a).

### TE frequency meta-analysis

The abundance of TEs near TSSs of P. patens and A. thaliana genes was assessed using publicly available genes and TEs annotations and a custom Perl script, which creates a histogram of scores relative to edges of entries from one annotation file based on the presence of entries from another annotation file. Gene annotations (v3.3 for *P. patens*, Araport11 for *Arabidopsis*) and *A. thaliana* TE annotation (TAIR10) were downloaded from www.phytozome.org. *P. patens* TE annotation^39^ was downloaded from www.genomevolution.org. TE annotations were reformatted to contain separate entries for start and end positions of each TE, and to assign each entry a score of 1. For each gene, the presence of TE edge was tested in a 25 bp sliding window up to 500 bp upstream to TSS, assigning positive windows with scores. In order to count only one edge of a TE closest to each gene, this analysis was performed separately on TEs ends against genes on the plus strand, and vice versa. Then, genes were aligned at TSS, and the percentages of genes with a TE ending in each 25 bp window were calculated.

### Percent methylation change

This number was calculated by dividing the difference in methylation level between two samples by the level of methylation in the sample with the higher methylation level. For example, percent-methylation-change between WT and cmt was calculated as follows:

1 $$\frac{{\mathrm{WT}}\, {\mathrm{mCHH}} - {\mathrm{cmt}}\, {\mathrm{mCHH}}}{{\mathrm{WT}}\, {\mathrm{mCHH}}} \times 100\qquad {\mathrm{if}}\ {\mathrm{WT}}\, {\mathrm{mCHH}} {\hskip 3pt} > {\hskip 3pt} {\mathrm{cmt}}\, {\mathrm{mCHH}}$$

2 $$- \frac{{\mathrm{cmt}}\, {\mathrm{mCHH}} - {\mathrm{WT}}\, {\mathrm{mCHH}}}{{\mathrm{cmt}}\, {\mathrm{mCHH}}} \times 100\qquad {\mathrm{if}}\ {\mathrm{WT}}\, {\mathrm{mCHH}} {\hskip 3pt} < {\hskip 3pt} {\mathrm{cmt}}\, {\mathrm{mCHH}}$$

### Box plots

Box plots compare percent-methylation-change within 50-bp windows with CHH methylation level of at least 0.1 in either of the samples, and with at least 20 informative sequenced cytosines. To examine the correlation between methylation change and chromatin structure, TE windows are separated into centiles in ascending order according to siRNA (24nt sRNA), GC ratio, H3K9me2, H3K4me3, TE size, and TE LTR/INT annotations. GC ratio and TE size were divided into five centiles. siRNA counts were divided into 10 centiles, which due to the high abundance of score 1 windows, only centile 1, 7, 9, and 10 are showing. H3K9me2 and H3K4me3 are Log2 ratio over total H3 that were divided into four centiles. For H3K9me2 and H3K4me3, we added an additional category, ND, that corresponded to windows that did not have any signal in either H3K9me2, H3K4me3 or H3.

### Identification of DMRs

Fractional methylation in 50 bp windows across the genome was compared between WT and each of the DNMT mutants. DMRs were called for windows with at least 0.1 fractional methylation, 10 informative sequenced cytosines, and Fisher’s exact test p-value <0.05.

### Reporting Summary

Further information on experimental design is available in the Nature Research Reporting Summary linked to this article.

## Supplementary information

Supplementary information

Reporting Summary

Source Data File

## Data Availability

The data supporting the findings of this study are available within the article and its Supplementary Information file. BS-seq data has been deposited in the Gene Expression Omnibus (GEO) database under the accession number GSE118153. The datasets generated and analyzed during the current study are available from the corresponding author on reasonable request. The source data underlying Fig. 1c is provided as a Source Data file. A reporting summary for this Article is available as a Supplementary Information file.
