# Publisher Correction: RdDM-independent de novo and heterochromatin DNA methylation by plant CMT and DNMT3 orthologs

**DOI:** 10.1038/s41467-019-10558-6

**Published:** 2019-06-06

**Authors:** Rafael Yaari, Aviva Katz, Katherine Domb, Keith D. Harris, Assaf Zemach, Nir Ohad

**Affiliations:** 1 School of Plant Sciences and Food Security, Tel-Aviv University, 69978 Tel- Aviv, Israel; 2 The Manna Center Program for Food Safety and Security, Tel Aviv University, 69978 Tel-Aviv, Israel

**Keywords:** Enzyme mechanisms, DNA methylation, Epigenomics, Plant evolution

Correction to: *Nature Communications* 10.1038/s41467-019-09496-0, published online 08 April 2019.

The original version of this Article contained an error in Fig. 5, in which the evolutionary origin of *DRM2* was incorrectly placed prior to the divergence between gymnosperms and angiosperms. The correct evolutionary origin of *DRM2* should be in angiosperms. The correct version of Fig. 5 is shown here as Fig. 1, which replaces the incorrect version which is shown here as Fig. 2. In addition, in the “Percent methylation change” section of the Methods, Equation 1 was incorrectly given as:

$$\frac{{\mathrm{WT}} \, {\mathrm{mCHH}} - {\mathrm{cmt}} \, {\mathrm{mCHHWT}} \, {\mathrm{mCHH}}}{\times} 100 \, {\mathrm{if}} \, {\mathrm{WT}} \, {\mathrm{mCHH}} \, > \, {\mathrm{cmt}} \, {\mathrm{mCHH}}$$

**Figure 1:**
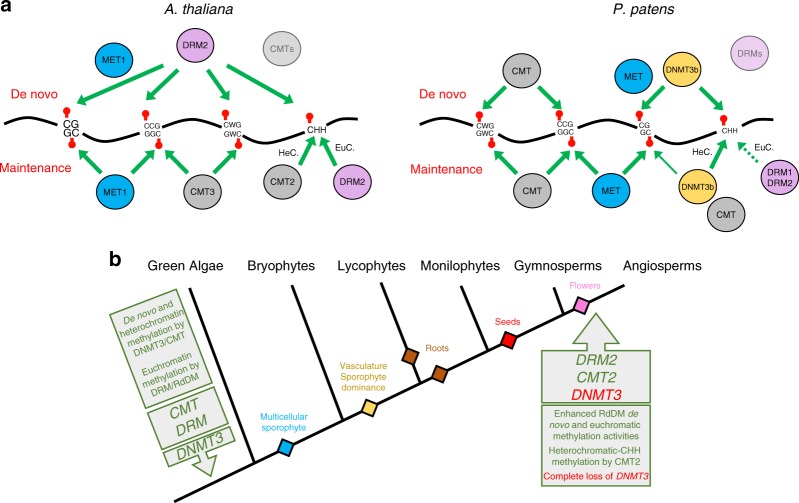
Fig. 1

**Figure 2:**
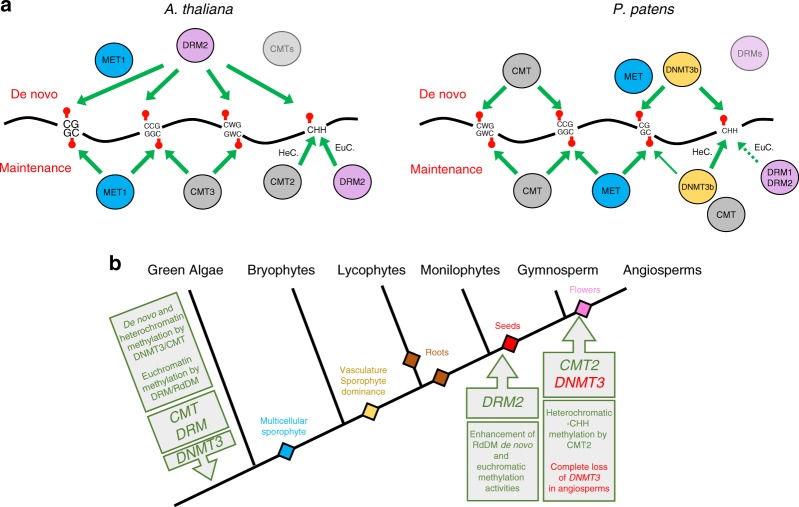
Fig.2

The correct form of Equation 1 is as follows:

1 $$\frac{{\mathrm{WT}} \, {\mathrm{mCHH}} - {\mathrm{cmt}} \, {\mathrm{mCHH}}}{{\mathrm{WT}} \, {\mathrm{mCHH}}} \times 100\quad\quad {\mathrm{if}} \, {\mathrm{WT}} \, {\mathrm{mCHH}} \, > \, {\mathrm{cmt}} \, {\mathrm{mCHH}}$$

This has been corrected in both the PDF and HTML versions of the Article.

