# Supplementary material for: RdDM-independent de novo and heterochromatin DNA methylation by plant CMT and DNMT3 orthologs
Source: Nat Commun. 2019 Apr 8;10:1613. doi: 10.1038/s41467-019-09496-0 (PMC6453930; doi:10.1038/s41467-019-09496-0)
Supplement: Supplementary file 1 — Supplementary information [file 41467_2019_9496_MOESM1_ESM.pdf]

RdDM-independent *de novo* and heterochromatin DNA methylation by plant CMT and DNMT3 orthologs

Yaari *et. al.*

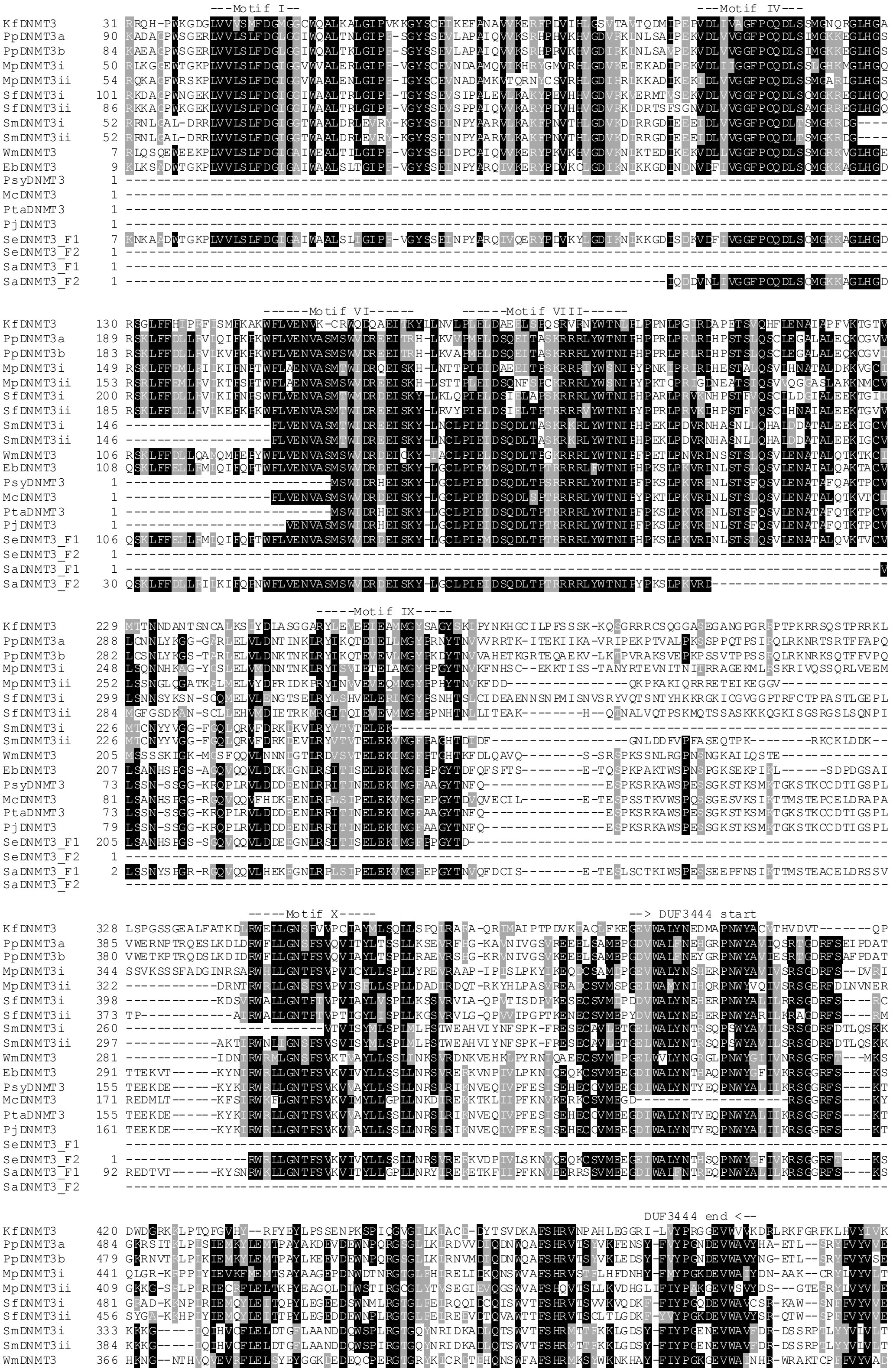

Supplementary Figure 1. Alignment of plant DNMT3 protein sequences. Sequences were aligned using MUSCLE [1]. Protein accessions are listed in Supplementary Table 1. Location of MTD motifs and the DUF3444 domain are denoted. Alignment printing format was generated with Boxshade 3.2.

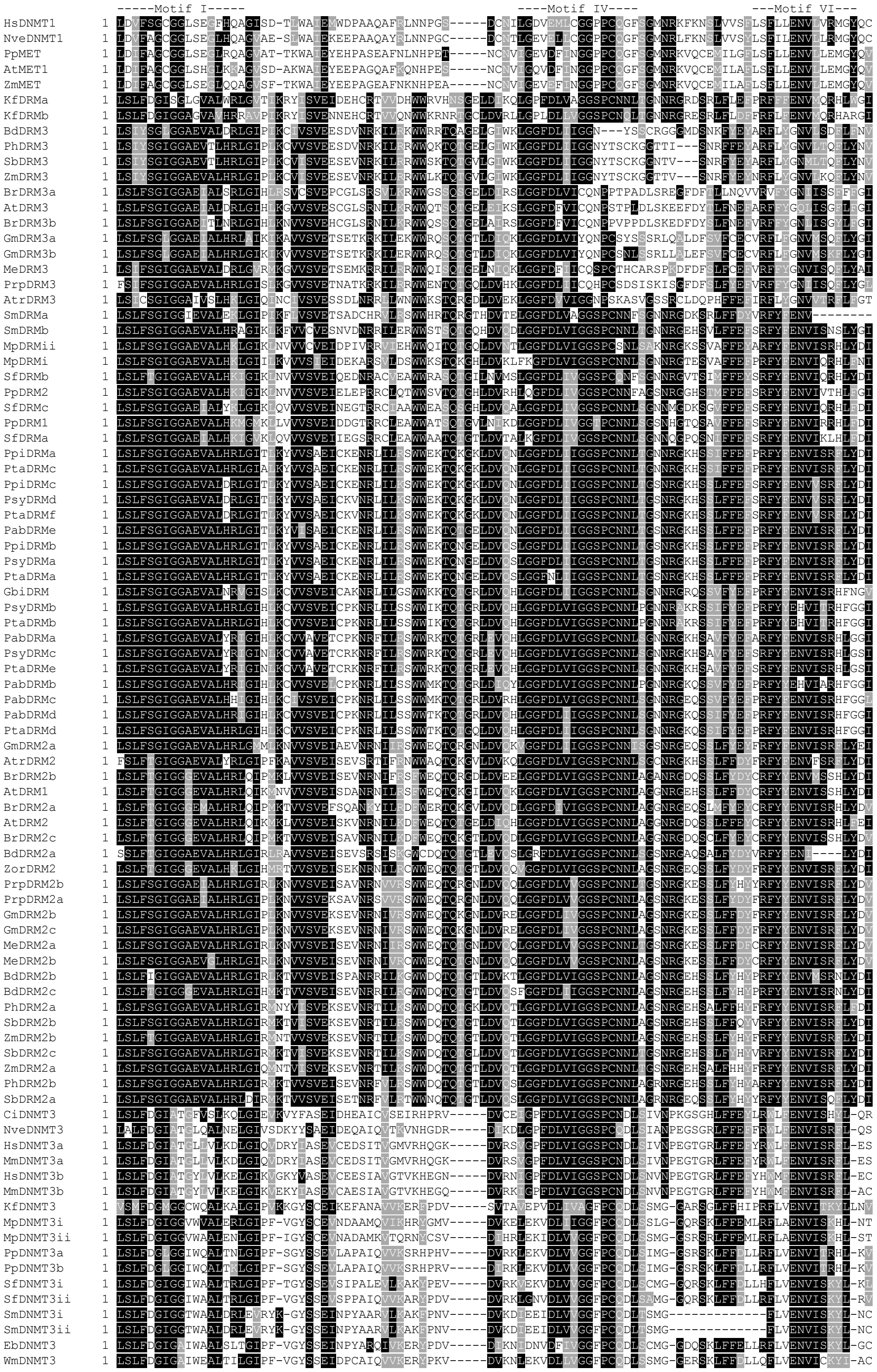
-----Motif I-----
----Motif IV----
---Motif VI---
HsDNMT1
NveDNMT1
PpMET
AtMET1
ZmMET
KfDRMa
KfDRMb
BdDRM3
PhDRM3
SbDRM3
ZmDRM3
BrDRM3a
AtDRM3
BrDRM3b
GmDRM3a
GmDRM3b
MeDRM3
PrpDRM3
AtrDRM3
SmDRMa
SmDRMb
MpDRMii
MpDRMi
SfDRMb
PpDRM2
SfDRMc
PpDRM1
SfDRMa
PpiDRMa
PtaDRMc
PpiDRMc
PsyDRMd
PtaDRMf
PabDRMe
PpiDRMb
PsyDRMa
PtaDRMa
GbiDRM
PsyDRMb
PtaDRMb
PabDRMa
PsyDRMc
PtaDRMe
PabDRMb
PabDRMc
PabDRMd
PtaDRMd
GmDRM2a
AtrDRM2
BrDRM2b
AtDRM1
BrDRM2a
AtDRM2
BrDRM2c
BdDRM2a
ZorDRM2
PrpDRM2b
PrpDRM2a
GmDRM2b
GmDRM2c
MeDRM2a
MeDRM2b
BdDRM2b
BdDRM2c
PhDRM2a
SbDRM2b
ZmDRM2b
SbDRM2c
ZmDRM2a
PhDRM2b
SbDRM2a
CiDNMT3
NveDNMT3
HsDNMT3a
MmDNMT3a
HsDNMT3b
MmDNMT3b
KfDNMT3
MpDNMT3i
MpDNMT3ii
PpDNMT3a
PpDNMT3b
SfDNMT3i
SfDNMT3ii
SmDNMT3i
SmDNMT3ii
EbDNMT3
WmDNMT3

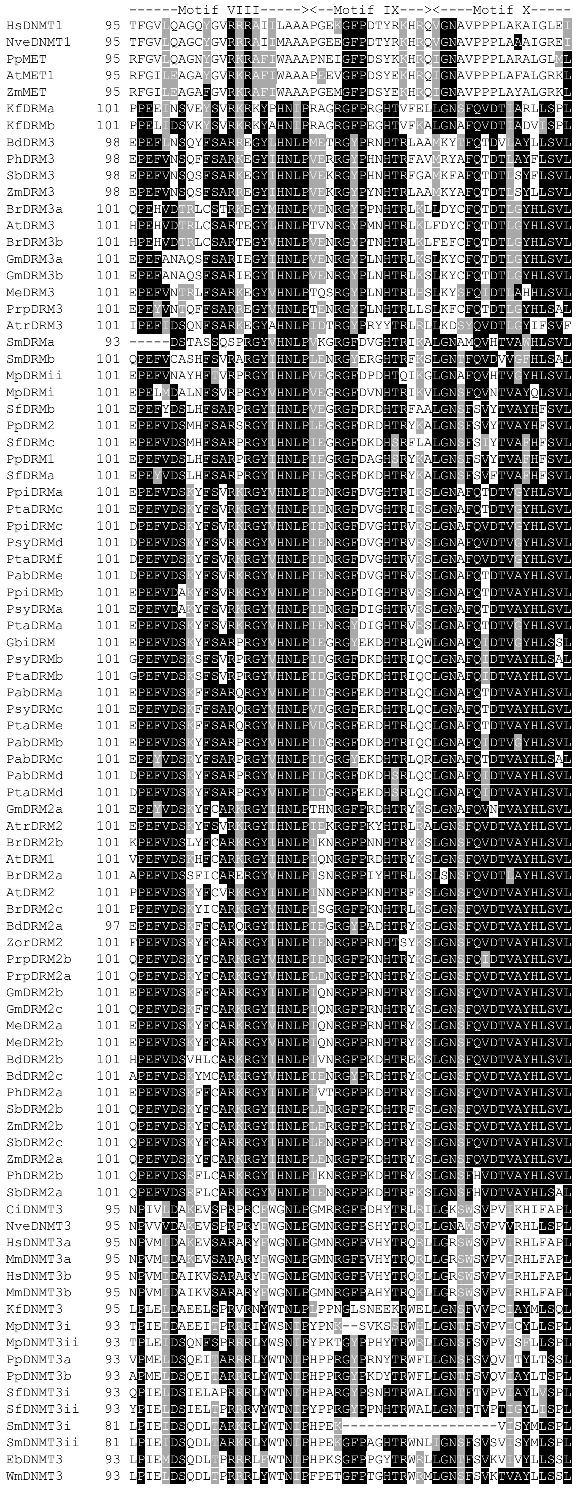

Supplementary Figure 2. Alignment of plant DNMT3 and DRM MTD motifs. Alignment of selected DNMT3, DRM and DNMT1 MTD protein sequences was performed using MUSCLE [1]. The motif order was rearranged in DRM sequences to match the linear organization of canonical DNMTs. For clear presentation, non-conserved blocks of alignment were filtered by Gblocks[2]. Protein accessions are listed in Supplementary Table 1. MTD motifs are denoted. Alignment printing format was generated with Boxshade 3.2.

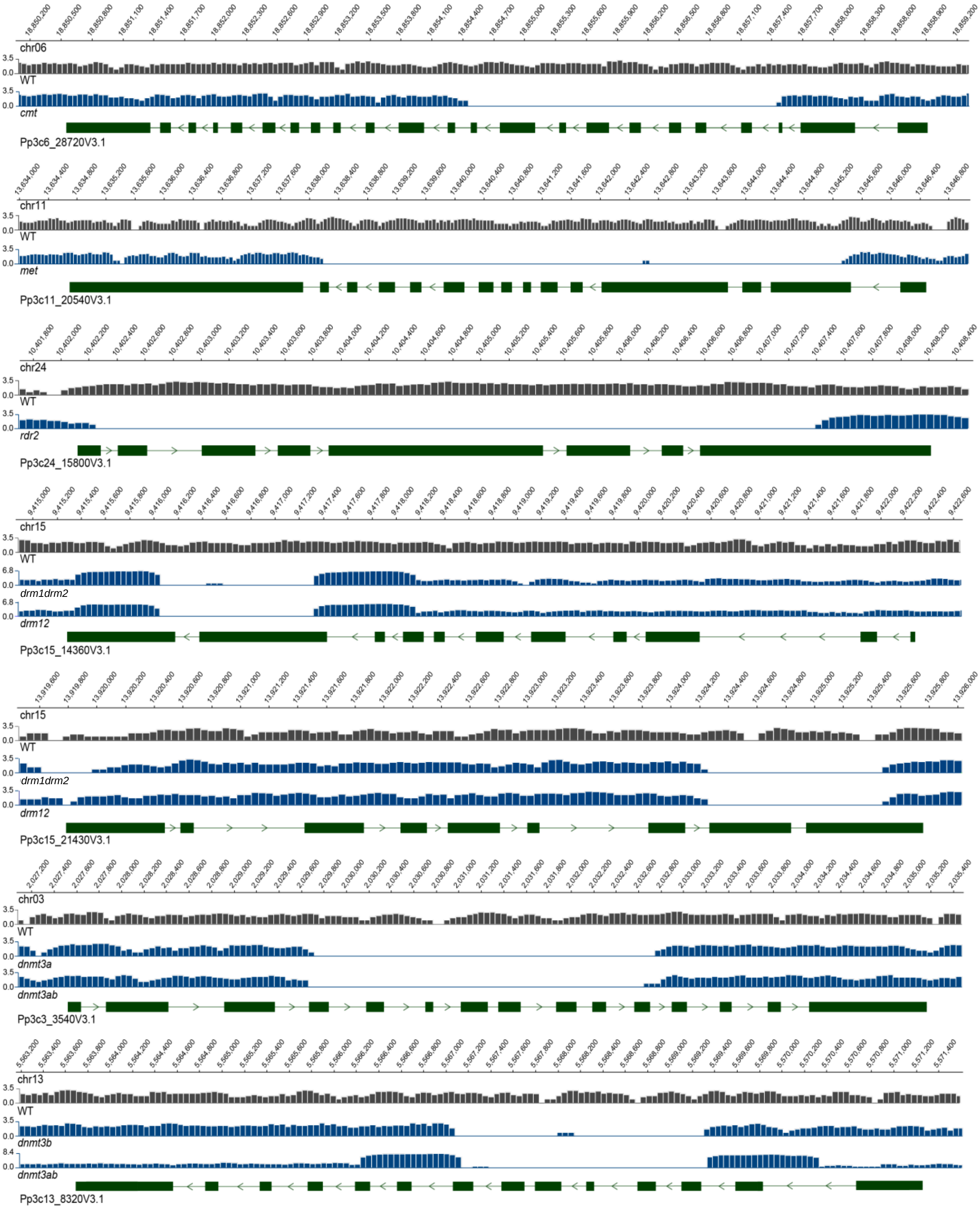

Supplementary Figure 3. Genotyping of PpDNMT mutants. Coverage of BS-seq reads at *PpDNMT* genes in WT and *dnmt* mutants.

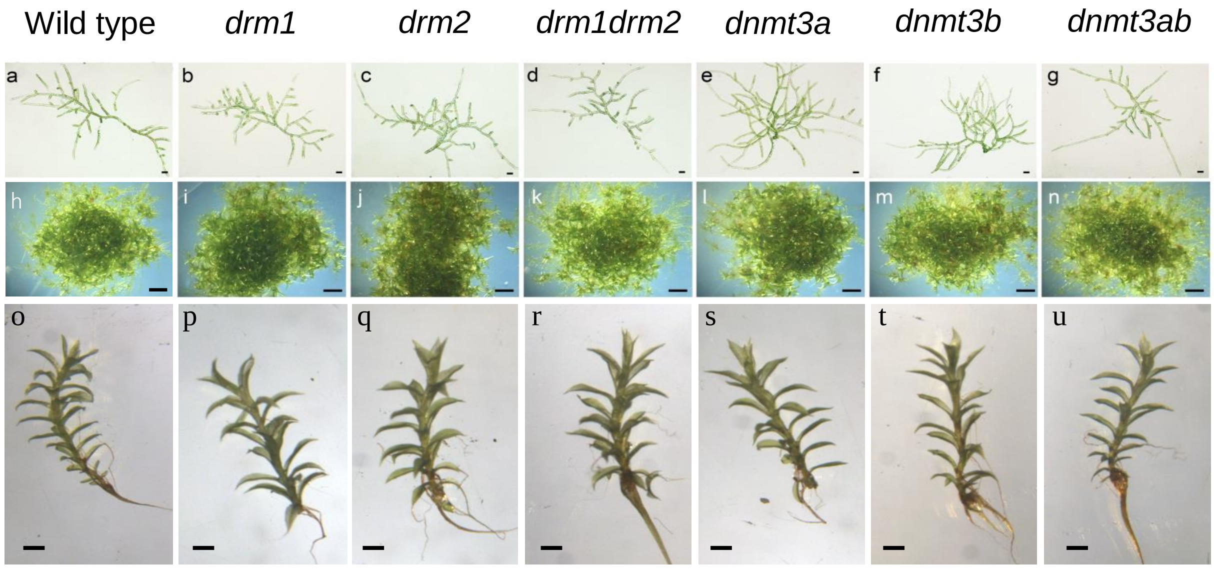

Supplementary Figure 4. Mutagenesis of PpDNMT3 and PpDRM does not disrupt *P. patens* development. Morphological analysis of protonema and gametophore development in WT and PpDNMT3 and DRM deletion mutants. **a-g**, Seven days old protonemata of WT (**a**) and mutants (**b-g**). Scale bar: 50 µm. **h-n**, Three weeks old plants bearing gametophores of WT (**h**) and mutants (**i-n**). Scale bar: 250 µm. **o-u**, Six weeks old gamethopores of WT (**o**) and mutants (**p-u**). Scale bar: 1 mm.

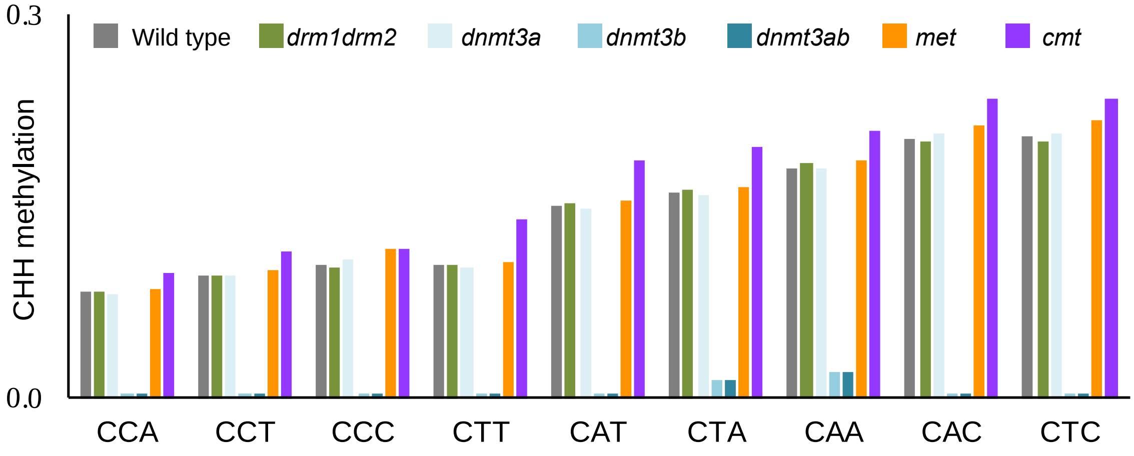

Supplementary Figure 5. Preferences in CHH methylation subcontexts. Averaged genomic CHH methylation level, in wild type and DNMT mutants, separated to its subcontexts.

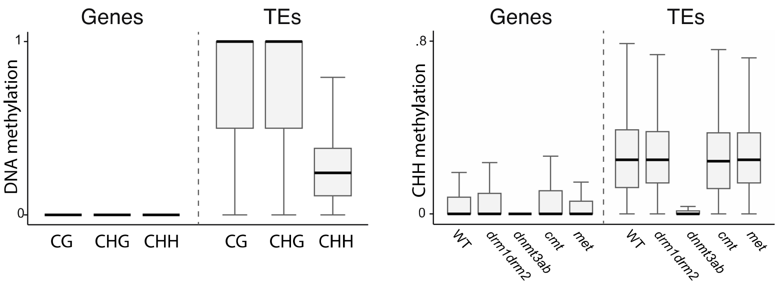

Supplementary Figure 6. DNA methylation in genes and transposons. Distribution of DNA methylation per 50 bp windows that either overlap with genes or with TEs. Left panel shows CG, CHG, and CHH methylation in wild type. Right panel shows CHH methylation in wild type and *dnmt* mutants within 50 bp windows that have at least 10% methylation in either of the samples.

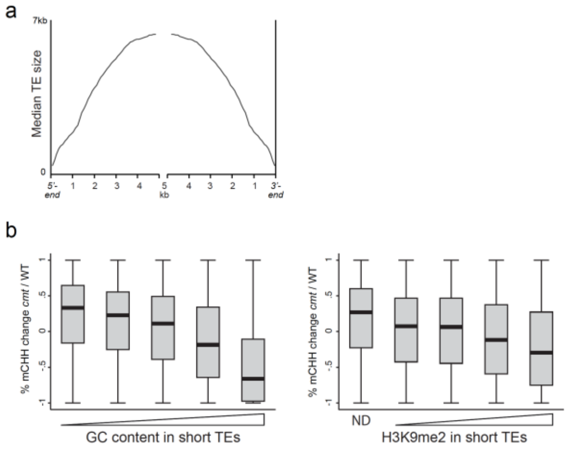

Supplementary Figure 7. Regulation of CHH methylation by PpCMT and PpDNMT3. a. Pattern of average TE size along the aligned TEs in Figures 2a, d, and 3e. *P. patens* TEs were aligned at the 5' or 3' ends and average TE size within each 100 bp interval is plotted. **b.** Box plots showing the distribution of percent-methylation-change per 50 bp windows between wild type and *cmt* mutant over H3K9me2, GC content within TEs shorter than 500 bp long.

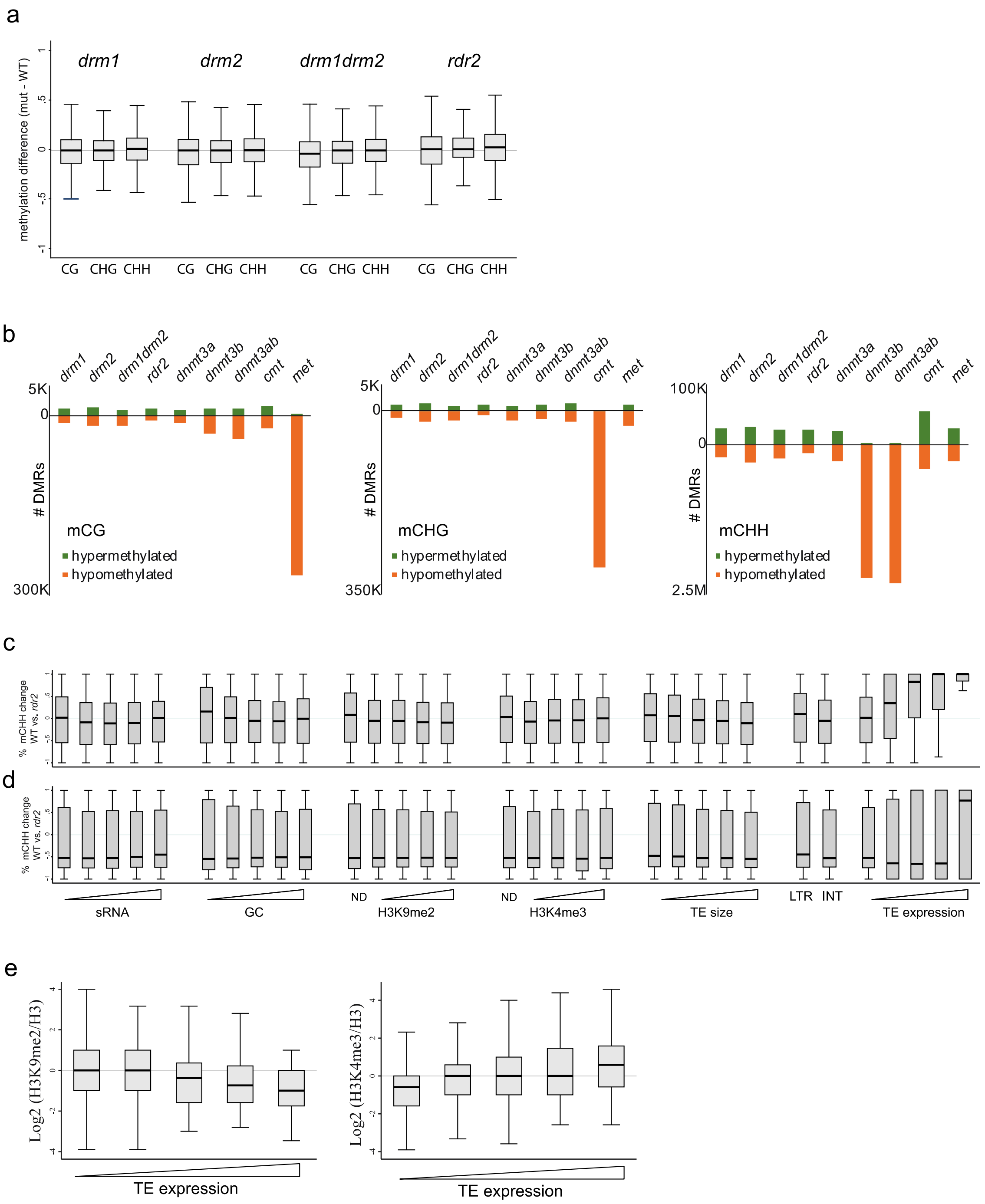

Supplementary Figure 8. DNA methylation in *drm* and *rdr2* mutants. a. DNA methylation difference between WT and indicated mutants. **b.** Number of hypo- and hyper-methylated CG, CHG, and CHH DMRs in each of the mutants. **c-d.** Percent-methylation-change between WT and *rdr2* mutant within *rdr2*-CHH-DMRs (c) or *drm12*-CHH-DMRs (d) over five centiles of indicated genomic or chromatin attributes. The WT plant is genetically unrelated to the *rdr2* one, thus comparison between these two plants could contribute to a noise level that could mask a weak hypo-methylation signal. Therefore, we analyzed the CHH methylation change in *rdr2* within *drm12*-CHH-DMRs (d). Note the change from global hypermethylation in *rdr2* in (c) to a slight hypo-methylation at low GC regions, short TEs, LTRs, and expressed TEs in (d).

Supplementary Table 1. DNMT gene model ID and source.

DNMT group	Name	Gene Model	Taxonomic Group1	Taxonomic Group2	Organism	Protein Length (AA)	Source
DNMT1	NveDNMT1	125496	Animals	Cnidaria	Nematostella vectensis	1263	Metazome
DNMT1	HsDNMT1	ENSG00000130816	Animals	Mammals	Homo sapiens	1632	Metazome
DNMT1	PpMET	Pp3c11_20540	Bryophytes	Mosses	Physcomitrella patens	1579	Phytozome
DNMT1	ZmMET	GRMZM2G333916_T01	Angiosperms	Monocots	Zea mays	1536	Phytozome
DNMT1	AtMET1	AT5G49160	Angiosperms	Eudicots	Arabidopsis thaliana	1535	Phytozome
DNMT3	NveDNMT3	127267	Animals	Cnidaria	Nematostella vectensis	624	Metazome
DNMT3	CiDNMT3	270939	Animals	Tunicata	Ciona intestinalis	618	Metazome
DNMT3	MmDNMT3a	ENSMUSP00000020991	Animals	Mammals	Mus musculus	910	Metazome
DNMT3	MmDNMT3b	ENSMUSP00000051830	Animals	Mammals	Mus musculus	860	Metazome
DNMT3	HsDNMT3a	ENSG00000119772	Animals	Mammals	Homo sapiens	914	Metazome
DNMT3	HsDNMT3b	ENSG00000088305	Animals	Mammals	Homo sapiens	853	Metazome
DNMT3	KfDNMT3	kfl00080_0350_v1.1	Charophytes		Klebsormidium flaccidum	893	K. nitens genome project
DNMT3	MpDNMT3i	Mapoly0043s0021.1	Bryophytes	Liverworts	Marchantia polymorpha	654	Phytozome
DNMT3	MpDNMT3ii	Mapoly0095s0030.1	Bryophytes	Liverworts	Marchantia polymorpha	687	Phytozome
DNMT3	PpDNMT3a	Pp3c3_3540V3.1	Bryophytes	Mosses	Physcomitrella patens	736	Phytozome
DNMT3	PpDNMT3b	Pp3c13_8320V3.1	Bryophytes	Mosses	Physcomitrella patens	711	Phytozome
DNMT3	SfDNMT3i	Sphfalx0068s0040.1	Bryophytes	Mosses	Sphagnum fallax	739	Phytozome
DNMT3	SfDNMT3ii	Sphfalx0127s0004.1	Bryophytes	Mosses	Sphagnum fallax	767	Phytozome
DNMT3	SmDNMT3i	fgenesh2_pg.C_scaffold_6000551	Lycophytes		Selaginella moellendorffii	630	Phytozome
DNMT3	SmDNMT3ii	fgenesh2_pg.C_scaffold_17000071	Lycophytes		Selaginella moellendorffii	685	Phytozome
DNMT3	EbDNMT3	GNQG-2005968	Gymnosperms	Cycadales	Encephalartos barteri	551	1KP
DNMT3	SeDNMT3_F1	KAWQ-2009204	Gymnosperms	Cycadales	Stangeria eriopus	248	1KP
DNMT3	SeDNMT3_F2	KAWQ-2054998	Gymnosperms	Cycadales	Stangeria eriopus	244	1KP
DNMT3	WmDNMT3	TOXE-2012823	Gymnosperms	Gnetales	Welwitschia mirabilis	562	1KP
DNMT3	PtaDNMT3	PTA00084430	Gymnosperms	Conifers	Pinus taeda	426	PLAZA
DNMT3	PsyDNMT3	PSY00011178	Gymnosperms	Conifers	Pinus sylvestris	426	PLAZA
DNMT3	McDNMT3	CDFR-2008757	Gymnosperms	Conifers	Manoao colensoi	405	1KP
DNMT3	SaDNMT3_F1	KLGF-2012241	Gymnosperms	Conifers	Sundacarpus amarus	344	1KP
DNMT3	SaDNMT3_F2	KLGF-2078876	Gymnosperms	Conifers	Sundacarpus amarus	105	1KP
DNMT3	PjDNMT3	MFTM-2078885	Gymnosperms	Conifers	Pinus jeffreyi	298	1KP
DRM	KfDRMa	kfl00018_0570_v1.1	Charophytes		Klebsormidium flaccidum	697	K. nitens genome project
DRM	KfDRMb	kfl00253_0110_v1.1	Charophytes		Klebsormidium flaccidum	1395	K. nitens genome project
DRM	MpDRMa	Mapoly0103s0053.3	Bryophytes	Liverworts	Marchantia polymorpha	723	Phytozome
DRM	MpDRMb	Mapoly0109s0015.2	Bryophytes	Liverworts	Marchantia polymorpha	758	Phytozome
DRM	PpDRM1	Pp3c15_14362	Bryophytes	Mosses	Physcomitrella patens	1036	Phytozome
DRM	PpDRM2	Pp3c15_21430	Bryophytes	Mosses	Physcomitrella patens	842	Phytozome
DRM	SfDRMa	Sphfalx0006s0094	Bryophytes	Mosses	Sphagnum fallax	551	Phytozome
DRM	SfDRMb	Sphfalx0011s0183	Bryophytes	Mosses	Sphagnum fallax	836	Phytozome
DRM	SfDRMc	Sphfalx0209s0004	Bryophytes	Mosses	Sphagnum fallax	790	Phytozome
DRM	SmDRMa	411110	Lycophytes		Selaginella moellendorffii	566	Phytozome
DRM	SmDRMb	fgenesh2_pg.C_scaffold_5000240	Lycophytes		Selaginella moellendorffii	551	Phytozome
DRM	GbiDRM	GBI00021934	Gymnosperms	Ginkgoales	Ginkgo biloba	655	PLAZA
DRM	PabDRMa	PAB00006266	Gymnosperms	Conifers	Picea abies	356	PLAZA
DRM	PabDRMb	PAB00012689	Gymnosperms	Conifers	Picea abies	482	PLAZA
DRM	PabDRMc	PAB00046707	Gymnosperms	Conifers	Picea abies	596	PLAZA
DRM	PabDRMd	PAB00048154	Gymnosperms	Conifers	Picea abies	532	PLAZA
DRM	PabDRe	PAB00049465	Gymnosperms	Conifers	Picea abies	398	PLAZA
DRM	PpiDRMa	PPI00065449	Gymnosperms	Conifers	Pinus pinaster	747	PLAZA
DRM	PpiDRMb	PPI00073543	Gymnosperms	Conifers	Pinus pinaster	423	PLAZA
DRM	PsyDRMa	PSY00004978	Gymnosperms	Conifers	Pinus sylvestris	747	PLAZA
DRM	PsyDRMb	PSY00005220	Gymnosperms	Conifers	Pinus sylvestris	473	PLAZA
DRM	PsyDRMc	PSY00006869	Gymnosperms	Conifers	Pinus sylvestris	662	PLAZA
DRM	PsyDRMd	PSY00008531	Gymnosperms	Conifers	Pinus sylvestris	645	PLAZA
DRM	PtaDRMa	PTA00004821	Gymnosperms	Conifers	Pinus taeda	687	PLAZA
DRM	PtaDRMb	PTA00013841	Gymnosperms	Conifers	Pinus taeda	581	PLAZA
DRM	PtaDRMc	PTA00015283	Gymnosperms	Conifers	Pinus taeda	367	PLAZA
DRM	PtaDRMd	PTA00023209	Gymnosperms	Conifers	Pinus taeda	461	PLAZA
DRM	PtaDRMe	PTA00046027	Gymnosperms	Conifers	Pinus taeda	435	PLAZA
DRM	PtaDRMf	PTA00067039	Gymnosperms	Conifers	Pinus taeda	349	PLAZA
DRM	AtrDRM3	evm_27.TU.AmTr_v1.0_scaffold00003.192	Angiosperms	Basel Angiosperms	Amborella trichopoda	719	Phytozome
DRM	AtrDRM2	evm_27.TU.AmTr_v1.0_scaffold00096.44	Angiosperms	Basel Angiosperms	Amborella trichopoda	503	Phytozome
DRM	BdDRM2a	Bradi1g11420	Angiosperms	Monocots	Brachypodium distachyon	518	Phytozome
DRM	BdDRM2b	Bradi1g77873	Angiosperms	Monocots	Brachypodium distachyon	610	Phytozome
DRM	BdDRM3	Bradi2g38577	Angiosperms	Monocots	Brachypodium distachyon	706	Phytozome
DRM	BdDRM2c	Bradi4g05680	Angiosperms	Monocots	Brachypodium distachyon	588	Phytozome
DRM	PhDRM3	Pahal.C01225	Angiosperms	Monocots	Panicum hallii	660	Phytozome
DRM	PhDRM2a	Pahal.I00079	Angiosperms	Monocots	Panicum hallii	603	Phytozome
DRM	PhDRM2b	Pahal.I00969	Angiosperms	Monocots	Panicum hallii	588	Phytozome
DRM	SbDRM2a	Sobic.001G458100	Angiosperms	Monocots	Sorghum bicolor	577	Phytozome
DRM	SbDRM2b	Sobic.001G535800	Angiosperms	Monocots	Sorghum bicolor	608	Phytozome
DRM	SbDRM2c	Sobic.003G124000	Angiosperms	Monocots	Sorghum bicolor	609	Phytozome
DRM	SbDRM3	Sobic.009G032200	Angiosperms	Monocots	Sorghum bicolor	657	Phytozome
DRM	ZmDRM3	GRMZM2G065599	Angiosperms	Monocots	Zea mays	461	Phytozome
DRM	ZmDRM2a	GRMZM2G092497	Angiosperms	Monocots	Zea mays	604	Phytozome
DRM	ZmDRM2b	GRMZM2G137366	Angiosperms	Monocots	Zea mays	610	Phytozome
DRM	ZorDRM3	Zosma21g00360.1	Angiosperms	Monocots	Zostera marina	597	Phytozome
DRM	ZorDRM2	Zosma67g00380.1	Angiosperms	Monocots	Zostera marina	570	Phytozome
DRM	AtDRM3	AT3G17310	Angiosperms	Eudicots	Arabidopsis thaliana	711	Phytozome
DRM	AtDRM2	AT5G14620	Angiosperms	Eudicots	Arabidopsis thaliana	627	Phytozome
DRM	AtDRM1	AT5G15380	Angiosperms	Eudicots	Arabidopsis thaliana	625	Phytozome
DRM	BrDRM2a	Brara.B00561	Angiosperms	Eudicots	Brassica rapa	406	Phytozome
DRM	BrDRM3b	Brara.E02454	Angiosperms	Eudicots	Brassica rapa	703	Phytozome
DRM	BrDRM2b	Brara.G02881	Angiosperms	Eudicots	Brassica rapa	599	Phytozome
DRM	BrDRM3a	Brara.I04643	Angiosperms	Eudicots	Brassica rapa	502	Phytozome
DRM	BrDRM2c	Brara.J02008	Angiosperms	Eudicots	Brassica rapa	602	Phytozome
DRM	GmDRM2a	Glyma.02G035700	Angiosperms	Eudicots	Glycine max	538	Phytozome
DRM	GmDRM2b	Glyma.05G005600	Angiosperms	Eudicots	Glycine max	591	Phytozome
DRM	GmDRM3a	Glyma.07G233200	Angiosperms	Eudicots	Glycine max	695	Phytozome
DRM	GmDRM3b	Glyma.17G038300	Angiosperms	Eudicots	Glycine max	730	Phytozome
DRM	GmDRM2c	Glyma.19G006100	Angiosperms	Eudicots	Glycine max	581	Phytozome
DRM	MeDRM3	Manes.03G210200	Angiosperms	Eudicots	Manihot esculenta	780	Phytozome
DRM	MeDRM2a	Manes.15G149300	Angiosperms	Eudicots	Manihot esculenta	639	Phytozome
DRM	MeDRM2b	Manes.17G113600	Angiosperms	Eudicots	Manihot esculenta	638	Phytozome
DRM	PrpDRM3	Prupe.1G109800	Angiosperms	Eudicots	Prunus persica	718	Phytozome
DRM	PrpDRM2a	Prupe.3G287400	Angiosperms	Eudicots	Prunus persica	585	Phytozome
DRM	PrpDRM2b	Prupe.8G038800	Angiosperms	Eudicots	Prunus persica	583	Phytozome

Supplementary Table 2. Primers used in this study.

	Primer Name	Sequence (5' -> 3')
**Cloning**	Ppdrm1 5'UTR BamHI Fw	GGATCCTGGAACGCAAAACAAGACCG
	Ppdrm1 5'UTR BamHI Rv	GGAGGTGGATCCAATTGTTCTTTC
	Ppdrm1 3'UTR SphI Fw	GCATGCTTCCCCTGGCAGAAATTTGG
	Ppdrm1 3'UTR NotI SphI Rv	GCATGCGGCCGCGAGCATCAAATTAGAGCTTCAGGG
	Ppdrm2 5'UTR BamHI Fw	GGATCCGTGGTGAACCTAGTTGTCCATTGG
	Ppdrm2 5'UTR BamHI Rv	GGATCCTGGCGTGTAAAGCTCACACTAA
	Ppdrm2 3'UTR SphI Fw	GCATGCGTTTGCCTTGCCTTGTTCCTTC
	Ppdrm2 3'UTR NotI SphI Rv	GCATGCGGCCGCCATCCTTTTGCAACAATCCTCC
	Ppdnmt3a HindIII 5'KO Fw	AAGCTTGTCGTGCTGAGTATTCAGATAATCGTAGC
	Ppdnmt3a HindIII 5'KO Rv	AAGCTTAATCCAACTGTTCCAATTCCGC
	Ppdnmt3a SphI 3'KO Fw	GCATGCTAGCTCTCTTGAAGTATCCG
	Ppdnmt3a 3'KO Rv	AATCGTGCTTTCTACCACATACTGCC
	Ppdnmt3b HindIII 5'KO Fw	AAGCTTCGGGTTTCGGAGTTCTGGGTT
	Ppdnmt3b HindIII 5'KO Rv	AAGCTTGCAGGCCAGAGGAAAGAGCG
	Ppdnmt3b SphI 3'KO Fw	GCATGCCCATGTTCCAATCTTTTGACTTGCC
	Ppdnmt3b 3'KO Rv	ACATTCCGTTTACCAGTAGCATCTGG
	KpnI Zeo Fw	GGTACCGTCAACATGGTGGAGCACGACA
	SphI Zeo Rv	GCATGCCAGGTCACTGGATTTTGGTTTTAGG
**Deletion mutant screening**	Ppdrm1 2240 5' Fw	GGAACACGGTGGATGTATTCCTTCT
	Ppdrm1 5550 3' Rv	AGGCGGTATGGTTGTGCCACC
	Ppdrm1 3209 e1 Fw	GGTCAAGGTCGAATCATCTCAACG
	Ppdrm1 3779 e1 Rv	GCGTTGGGATGTTTGGAGCA
	Ppdrm2 2292 5' Fw	GACAATTTCCATTCATGCGAGTTGTC
	Ppdrm2 5324 3' Rv	CAAGCCATGCCTATTGTTATCACTGTTC
	Ppdrm2 3202 e1 Fw	ATTGGCTTTGGTCTTCCTGGTCA
	Ppdrm2 3581 e1 Rv	TGTGGGAATTGCAGTGGCGT
	Ppdnmt3a 5046 Fw	GCTGCAAGCGTGAGCGATTC
	Ppdnmt3a 10651 Rv	GGGTTGGATATCACTAAGCTCCACC
	Ppdnmt3a 6377 Fw	GCTGACCAATCTAGGCATCCCG
	Ppdnmt3a 8427 Rv	TGGAGGGCTTGATTTAGGCAGAG
	Ppdnmt3b 5678 Fw	GCTGATGACTGCTTGAGCCTTCG
	Ppdnmt3b 10445 Rv	TCCACTCGTCTACTTCTTCTTTGAGATAGG
	Ppdnmt3b 7159 Fw	GGTCGGGTGAACGGCTGG
	Ppdnmt3b e11 Rv	AAGGCTATCCTGTCGAGTTGGCTT
	35S Rv	TGGGACCACTGTCGGCAGAG
	35S-Ter-R-Fw	GCCCCCGCTTAAAAATTGGT
**Validation of RPS insertion**	RPS-top-R_new	AAGTAGAGAAAGGAAAGAGAAAAGGGG
	35S Rv	TGGGACCACTGTCGGCAGAG
**Bisulfite sequencing (Sanger)**	RPS-top-R_new	AAGTAGAGAAAGGAAAGAGAAAAGGGG
	RPS-top-F	CTGTATTTTTCTCCCTTCA
**BS-seq**	bs-seq-adapter-fwd	GATCGGAAGAGCGGTTCAGCAGGAATGCCGA*G
	bs-seq-adapter-rv	ACACTCTTTCCCTACACGACGCTCTTCCGATC*T
	bs-seq-primer_fwd	AATGATACGGCGACCACCGAGATCTACACTCTTTCCCTACACGACGCTCTTCCGATC*T
	bs-seq-primer-rv	CAAGCAGAAGACGGCATACGAGATCGGTCTCGGCATTCCTGCTGAACCGCTCTTCCGATC*T
		*= phosphorothioate bond, C= methylated cytosine

Supplementary Table 3. BS-seq stats summary.

Genetic background	Mapping			Median site coverage	Average genomic methylation levels				Average organelle methylation levels	
	Total reads	Mapped reads	Ratio		CG	CWG	CCG	CHH	Mitochondria	Chloroplast
wild type	41145982	34088890	83%	3	19.56%	21.12%	8.44%	15%	0.17%	0.17%
drm1	41372443	33415124	81%	3	20.9%	22.26%	9.57%	16.24%	0.17%	0.17%
drm2	52331188	42380753	81%	3	19.79%	21.25%	8.87%	15.29%	0.17%	0.17%
drm12	39059321	31104455	80%	3	19.43%	21.18%	8.84%	15.38%	0.17%	0.16%
dnmt3a	46395247	34361843	74%	3	20.14%	21.4%	8.83%	14.88%	0.17%	0.17%
dnmt3b	46037700	34325065	75%	3	19.94%	22.79%	10.68%	0.84%	0.18%	0.17%
dnmt3ab	51456246	40940817	80%	3	19.62%	22.59%	10.57%	0.83%	0.17%	0.16%
met	48204209	37218804	77%	3	1.28%	21.74%	0.65%	15.35%	0.17%	0.17%
cmt	47855012	37225542	78%	3	20.68%	0.49%	0.39%	15.5%	0.17%	0.17%
rdr2	51728794	40797730	79%	3	24.8%	25.83%	13.2%	17.82%	0.53%	0.59%

Median coverage and averaged methylation levels in wild type and PpDNMT mutant genomes. Substantial loses in methylation (>90%) are marked in red.

Supplementary References

1. Edgar, R. C. MUSCLE: multiple sequence alignment with high accuracy and high throughput. *Nucleic Acids Res* **32,** 1792–1797 (2004).
2. Castresana, J. Selection of conserved blocks from multiple alignments for their use in phylogenetic analysis. *Mol. Biol. Evol.* **17,** 540–552 (2000).
