# Supplementary material for: RdDM-independent de novo and heterochromatin DNA methylation by plant CMT and DNMT3 orthologs
Source: Nat Commun. 2019 Apr 8;10:1613. doi: 10.1038/s41467-019-09496-0 (PMC6453930; doi:10.1038/s41467-019-09496-0)
Supplement: Supplementary file 2 — Reporting Summary [file 41467_2019_9496_MOESM2_ESM.pdf]

nature research

Corresponding author(s): Assaf Zemach and Nir Ohad

Reporting Summary

Nature Research wishes to improve the reproducibility of the work that we publish. This form provides structure for consistency and transparency in reporting. For further information on Nature Research policies, see Authors & Referees and the Editorial Policy Checklist.

Statistical parameters

When statistical analyses are reported, confirm that the following items are present in the relevant location (e.g. figure legend, table legend, main text, or Methods section).

n/a	Confirmed	
☐	☒	The exact sample size (*n*) for each experimental group/condition, given as a discrete number and unit of measurement
☐	☒	An indication of whether measurements were taken from distinct samples or whether the same sample was measured repeatedly
☐	☒	The statistical test(s) used AND whether they are one- or two-sided<br>*Only common tests should be described solely by name; describe more complex techniques in the Methods section.*
☐	☒	A description of all covariates tested
☐	☒	A description of any assumptions or corrections, such as tests of normality and adjustment for multiple comparisons
☐	☒	A full description of the statistics including central tendency (e.g. means) or other basic estimates (e.g. regression coefficient) AND variation (e.g. standard deviation) or associated estimates of uncertainty (e.g. confidence intervals)
☐	☒	For null hypothesis testing, the test statistic (e.g. *F*, *t*, *r*) with confidence intervals, effect sizes, degrees of freedom and *P* value noted<br>*Give P values as exact values whenever suitable.*
☒	☐	For Bayesian analysis, information on the choice of priors and Markov chain Monte Carlo settings
☒	☐	For hierarchical and complex designs, identification of the appropriate level for tests and full reporting of outcomes
☐	☒	Estimates of effect sizes (e.g. Cohen's *d*, Pearson's *r*), indicating how they were calculated
☒	☐	Clearly defined error bars<br>*State explicitly what error bars represent (e.g. SD, SE, CI)*

Our web collection on statistics for biologists may be useful.

Software and code

Policy information about availability of computer code

Data collection	blast https://blast.ncbi.nlm.nih.gov/Blast.cgi
Data analysis	blast https://blast.ncbi.nlm.nih.gov/Blast.cgi<br>MUSCLE v3.8.31<br>Boxshade v3.2<br>IQ-TREE v1.6.4<br>FigTree v1.4.3<br>Bowtie2 v2.3.2<br>R v3.3.1 via Rstudio v1.0.136<br>STATA /SE v14.2<br>IGV genome browser v2.3<br>SignalMap v2.0<br>bs-sequel.pl (a custom batch of perl scripts)<br>correlateSingleEnds.pl (a custom perl script)<br>collect_align_stats.pl (a custom perl script)<br>countMethylation.pl (a custom perl script)<br>fisher_exact_test.pl (a custom perl script)<br>gff-methyl-stats.pl (a custom perl script)

armageddon_analysis.pl (a custom perl script)
Venn Diagram Plotter v1.5.5228
KaleidaGraph v4.0
Adobe Illustrator 2015.3.0

For manuscripts utilizing custom algorithms or software that are central to the research but not yet described in published literature, software must be made available to editors/reviewers upon request. We strongly encourage code deposition in a community repository (e.g. GitHub). See the Nature Research guidelines for submitting code & software for further information.

Data

Policy information about availability of data

All manuscripts must include a data availability statement. This statement should provide the following information, where applicable:

- Accession codes, unique identifiers, or web links for publicly available datasets
- A list of figures that have associated raw data
- A description of any restrictions on data availability

Sequencing data have been deposited in Gene Expression Omnibus under accession number GSE116837

Field-specific reporting

Please select the best fit for your research. If you are not sure, read the appropriate sections before making your selection.

☒ Life sciences ☐ Behavioural & social sciences ☐ Ecological, evolutionary & environmental sciences

For a reference copy of the document with all sections, see nature.com/authors/policies/ReportingSummary-flat.pdf

Life sciences study design

All studies must disclose on these points even when the disclosure is negative.

Sample size

Sample size was considered for the RPS methylation experiment. Finding a normal methylation pattern (in all contexts CG, CHG, and CHH) in 19 to 22 RPS molecules that originate from three independent transgenic lines on the background of each drm1/drm2 and dnmt3a mutants is sufficient to claim that de novo methylation can be performed in the absence of PpDRMs or PpDNMT3a (as even a single methylated molecule is sufficient to make that claim). The methylation effect in dnmt3b, dnmt3ab, cmt, and met, is based on 19 to 31 individually-cloned PCR fragments. The context-specific methylation effect, i.e. CG/CHH in dnmt3b, CHG in cmt, and CG in met, substantially reduces the chance for technical bisulfite conversion or PCR biases, and thus allow to safely claim that these DNMTs are crucial for the methylation of the RPS transgene, each DNMT in a particular sequence contexts.

Data exclusions

No data were excluded from the analysis

Replication

P. patens DNMTs deletion mutants were generated by replacing the genomic region coding for their methyltransferase domain with a resistance cassette via homologous recombination, thus considered as complete dnmt null mutants.

RPS methylation:
RPS methylation was profiled from at least 19 individually-cloned PCR fragments per each examined dnmt mutant. RPS methylation of rdr2 mutant was profiled using BS-seq.

Genomic methylation:
Single replicates for Ppmet, Ppcmt, and Ppdnmt3b showed a near complete genomic hypomethylation in a context specific manner, implying for a real signal that cannot be influenced by either biological or technical noise. Ppdnmt3b genomic CHH hypomethylation phenotype confirmed in the double dnmt3a/dnmt3b mutant. Ppdrm1 and Ppdrm2 trivial methylation phenotypes was confirmed in the double drm1/drm2 mutant as well as in the rdr2 mutant. BS-seq genomic methylation profiles were independently confirmed for all dnmt mutants, single and double, using traditional bisulfite-PCR and sanger sequencing and can be provided per reviewer request.

Randomization

P. patens plants, wild type and dnmt mutants, were randomly placed in the growing chambers. DNA extractions, library preparations, and sequencing were performed simultaneously to all samples.

Blinding

Except for rdr2 mutant, prior to DNA extraction, each of the dnmt mutant and wild type samples was labeled with a code that was known only to the experimentalist and not to the data analyst. Matching between global methylation phenotype and genetic background (i.e. Fig 1b) was done post analysis. All downstream methylation data analyses were unblinded.

Reporting for specific materials, systems and methods

Materials & experimental systems

n/a	Involved in the study	
☐	☒	Unique biological materials
☒	☐	Antibodies
☒	☐	Eukaryotic cell lines
☒	☐	Palaeontology
☒	☐	Animals and other organisms
☒	☐	Human research participants

Methods

n/a	Involved in the study	
☒	☐	ChIP-seq
☒	☐	Flow cytometry
☒	☐	MRI-based neuroimaging

Unique biological materials

Policy information about availability of materials

Obtaining unique materials	Transgenic plants generated in this study are readily available from the authors.
